# Quality of maternal health services in Lagos, Nigeria: Women’s perspectives across healthcare districts

**DOI:** 10.1371/journal.pone.0359548

**Published:** 2026-11-06

**Authors:** Jonathan Odumegwu, Adedayo Aderibigbe, Onoseta Momodu, Ozi Obiamalu, Ayokunle Olumodeji, Precious Tettehfio, Folashade Oludara, Melody Ucho, Joyce Gyamfi, Michael Amegbor, Emmanuel Peprah, Ololade Wright

**Affiliations:** 1 Implementing Sustainable Evidence-based interventions through Engagement (ISEE) Lab, New York University (NYU) School of Global Public Health, NYU School of Global Public Health, New York, New York, United States of America; 2 Department of Statistical Sciences, Baylor University, Waco, Texas, United States of America; 3 Department of Community Health and Primary Health Care, Lagos State University Teaching Hospital (LASUTH), Ikeja, Lagos, Nigeria; 4 Department of Obstetrics and Gynaecology, Lagos State University Teaching Hospital (LASUTH), Ikeja, Lagos, Nigeria; 5 Clean Fuel – Cookstove (CF-CS) Lab, Lagos State University College of Medicine (LASUCOM), Ikeja, Lagos, Nigeria; 6 Family Health Directorate, Lagos State Ministry of Health, Alausa, Lagos, Nigeria; 7 New York University (NYU) School of Global Public Health, NYU School of Global Public Health, New York, New York, United States of America; 8 Department of Community Health and Primary Health Care, Lagos State University College of Medicine (LASUCOM), Ikeja, Lagos, Nigeria; Innovative Aid, CANADA

## Abstract

**Background:**

Women’s perceptions of care quality during maternal health visits influence service use and maternal and neonatal outcomes. This study assesses perceived quality of maternal health services across healthcare districts in Lagos, Nigeria.

**Methods:**

This cross-sectional study analyzed data from 7,324 women aged 15–49 years in Lagos State from the 2022 LARCHIS Survey. It examined how demographic, reproductive, care-use, and facility factors predicted perceived maternal healthcare quality (poor/fair, good, or excellent) using multinomial logistic and mixed-effects models.

**Results:**

Among 7,324 women (mean age: 31.2 ± 5.9 years), perceived service quality varied significantly by district and facility type. Compared with District I, women in District III were less likely to rate services as good (aOR = 0.37; 95% CI: 0.24–0.56) or excellent (aOR = 0.08; 95% CI: 0.05–0.14), whereas women in District V had higher odds. Lack of response to inquiries was associated with lower odds of rating services as good (aOR = 0.12; 95% CI: 0.08–0.18) or excellent (aOR = 0.06; 95% CI: 0.03–0.16). Rude staff behavior similarly reduced the odds of good ratings (aOR = 0.15; 95% CI: 0.11–0.21) and excellent ratings (aOR = 0.06; 95% CI: 0.06–0.13). Missionary and private facilities received higher ratings than public facilities. Facility readiness—24-hour services, doctor availability, and skilled staff—predicted higher ratings, whereas long waits and closures predicted lower ratings. Staff courtesy and responsiveness were the strongest predictors.

**Conclusion:**

Women’s perceptions of maternal healthcare in Lagos are shaped by structural and interpersonal factors. Improving facility readiness, reducing delays, and promoting respectful care may improve experiences, service use, and maternal and neonatal outcomes.

## Introduction

Maternal mortality remains a persistent global health challenge, despite longstanding international commitments to reduce it [1]. An estimated 260,000 maternal deaths occurred worldwide in 2023 [2,3], with sub-Saharan Africa bearing a disproportionate burden, accounting for nearly 70% of maternal deaths in low- and middle-income countries [1]. Nigeria contributes significantly to this toll [4,5], representing approximately 28.7% of all estimated global maternal deaths in 2023, with a maternal mortality ratio (MMR) of 993 per 1000,000 live births and an estimated 75,000 deaths [1,6].

The Sustainable Development Goals (SDG) 3.1 target a global MMR of less than 70 per 100,000 live births by 2030, with no country or state expected to exceed 140 per 100,000 live births [7,8]. While Nigeria has shown modest progress in MMR, declining from over 800 maternal deaths per 100,000 live births in 2015 to approximately 512 in 2018, this improvement has been uneven across states and populations [1,9,10]. Lagos State, Nigeria’s most urbanized and populous state, presents a significant paradox. Despite extensive public investments in health infrastructure and maternal services, including health care facilities, maternal mortality has remained persistently high [11,12]. A recent subnational estimate reported an MMR of 430 deaths per 100,000 live births in Lagos State, nearly unchanged from a decade earlier [10]. This stagnation exemplifies the urban health penalty, in which marginalized populations in megacities fail to benefit from the presumed urban advantage of better health care access [12–15].

The slow progress in reducing maternal mortality underscores systemic inequities in access to and quality of maternal care [7]. While previous studies have largely focused on clinical causes of maternal deaths, such as hemorrhage, hypertensive disorders, or sepsis, fewer have examined the socio-structural and perceptual factors that shape women’s health-seeking behavior. Evidence from Nigeria and other sub-Saharan African settings shows that poor perceived quality of care, disrespectful treatment, inadequate drug supplies, obsolete medical equipment, and overcrowded facilities can deter women from delivering in health facilities [14,16,17]. Primary health centers, which serve as the entry point for most women, are often constrained by staffing shortages and inconsistent adherence to clinical standards. By contrast, secondary and tertiary facilities face overcrowding and long waiting times that compromise patient-centered, respectful care [12,16,18–20].

Women’s perceptions of care are therefore critical [20,21]. The World Health Organization’s quality-of-care framework emphasizes not only evidence-based clinical practices and service availability but also dignity, respect, and person-centered care [21]. Women’s experiences during health care visits shape their trust in the health system, adherence to medical advice, and willingness to seek skilled care in future pregnancies [22,23]. Studies from Kenya, Tanzania, and Ghana confirm that both objective access (e.g., distance, cost, road conditions) and subjective quality (e.g., provider communication, cleanliness, interpersonal respect) determine maternal health care utilization [13,15,16,24–26]. In Nigeria, reports of disrespect and abuse during maternal health care visits and childbirth further discourage facility-based deliveries, perpetuating the cycle of preventable maternal deaths [17,27,28].

Despite a growing body of literature on maternal healthcare quality in Nigeria and sub-Saharan Africa, important gaps remain. Much of the existing evidence is derived from facility-based studies, often limited to tertiary centers [29], which may not reflect the experiences of women across different levels of care or geographic contexts. Prior studies have often focused on clinical indicators or service utilization, with limited attention to women’s subjective experiences of care, which are increasingly recognized as critical components of health system performance [30,31]. Most existing research on maternal perceptions of care in Nigeria is drawn from hospital-based studies, often conducted at tertiary centers, which may not fully capture the realities of women who deliver or seek care outside licensed or accredited public and private primary, secondary, and tertiary health care facilities [10,12]. There have been limited studies and gaps in identifying potential factors that attenuate perceptions of care quality among women across Lagos State’s health districts, where the enactment of preventive policies, strategies, and early intervention could be most effective. In addition, no studies have used large, population-based datasets to examine both structural and interpersonal determinants of perceived quality across the newly created health districts in Lagos State.

Quality of care varies substantially across primary, secondary, and tertiary health care facilities [22]. Primary health facilities, though most accessible and usually government-owned, are often understaffed and inadequately supplied, while higher-level facilities struggle with long queues and limited privacy, eroding women’s experiences of respectful care [21,22]. Using large population-based datasets across Lagos State’s health districts to investigate factors shaping maternal perceptions of care quality and adherence to health care-seeking would help identify areas requiring urgent stakeholder interventions to improve the quality of maternal health care.

Given the gaps in the literature, understanding of how newly established health districts in Lagos State improve maternal health and shape women’s perceptions of care quality, particularly among women of reproductive age, remains limited. Therefore, the present study aims to address these gaps by determining whether there are significant differences in perceptions of maternal care quality across health districts and in relation to various interpersonal factors (such as age, age at marriage, ethnicity, education level, and marital status), and health and structural factors (including use of a healthcare facility, loss of an infant, and the number of ANC sessions attended). By examining these factors, this study, using the Lagos State Reproductive and Child Health Indicator Survey (LARCHIS) conducted in 2022, which offers a rare population-based lens on maternal perceptions of care across all twenty local government areas and six health districts in Lagos State, Nigeria, and seeks to clarify how structural, health, and interpersonal factors shape maternal health and to identify critical points for prevention and intervention.

## Methods

### Study setting

Lagos State, located in southwestern Nigeria, is administratively divided into five divisions and 20 Local Government Areas (LGAs) (Table 1), which are further subdivided into wards and enumeration areas (EAs). The state also comprises six recently created Healthcare Districts responsible for healthcare administration.

**Table 1:** Lagos state healthcare districts.

HealthCare District I	Agege LGA, Alimosho LGA, and Ifako-Ijaiye LGA
HealthCare District II	Ikorodu LGA, Kosofe LGA, Shomolu LGA
HealthCare District III	Epe LGA, Eti-Osa LGA and Ibeju-Lekki LGA
HealthCare District IV	Apapa LGA, Surulere LGA, Lagos Island LGA and Lagos Mainland LGA
HealthCare District V	Badagry LGA, Ojo LGA and Amuwo-Odofin LGA and Ajeromi-Ifelodun
Health Care District VI	Oshodi-Isolo LGA, Mushin LGA and Ikeja LGA

### Study design and sampling

Our study is a cross-sectional analysis of secondary data from the LARCHIS project, a state-wide survey conducted from April to August 2022 across all 20 LGAs and 6 Healthcare Districts of Lagos State, Nigeria (Table 1). The primary study targeted women of reproductive age (15–49 years) and aimed to estimate key maternal and child health indicators. The LARCHIS employed a multi-stage cluster sampling design to ensure representativeness across all healthcare districts in Lagos State. Enumeration areas were selected using probability proportional to size, followed by systematic sampling of households. Where applicable, design effects and sampling weights were applied to account for unequal selection probabilities and enhance representativeness [5]. Data were collected using a pre-tested, semi-structured, interviewer-administered questionnaire adapted from the Nigeria Demographic and Health Survey (NDHS) and the Multiple Indicator Cluster Survey (MICS). In addition to demographic and population indicators, the questionnaire collected information on access to maternal and child health services and participants’ perceptions of healthcare quality based on personal experiences. Although this study addressed a different research question from the original survey, the dataset’s breadth and variability provided sufficient scope for rigorous secondary analysis. We analyzed de-identified data, and no new data were collected or participants enrolled.

### Analytic sample

A total of 10,000 women participated in the 2022 LARCHIS (Fig 1). A complete-case analysis approach was used, and records with missing data on key variables were excluded. The proportion and distribution of missing data were assessed to minimize potential bias. We excluded 394 participants (4.0%) who had not given birth. Among the remaining respondents (n = 9,606), 2,282 records (23.8%) were excluded due to missing data on key variables: educational status (n = 5), history of using medical facilities (n = 1), having given birth alive but later died (n = 44), type of healthcare worker at delivery (n = 910), antenatal visits (n = 44), type of facility used (n = 1,183), ethnicity (n = 6), all sisters (n = 80), and marital status (n = 9). In addition, of the 7,324 complete records, 93 respondents who had never used community facilities were further excluded, resulting in a final analytic sample of 7,231 women (Fig 1).

**Fig 1:**
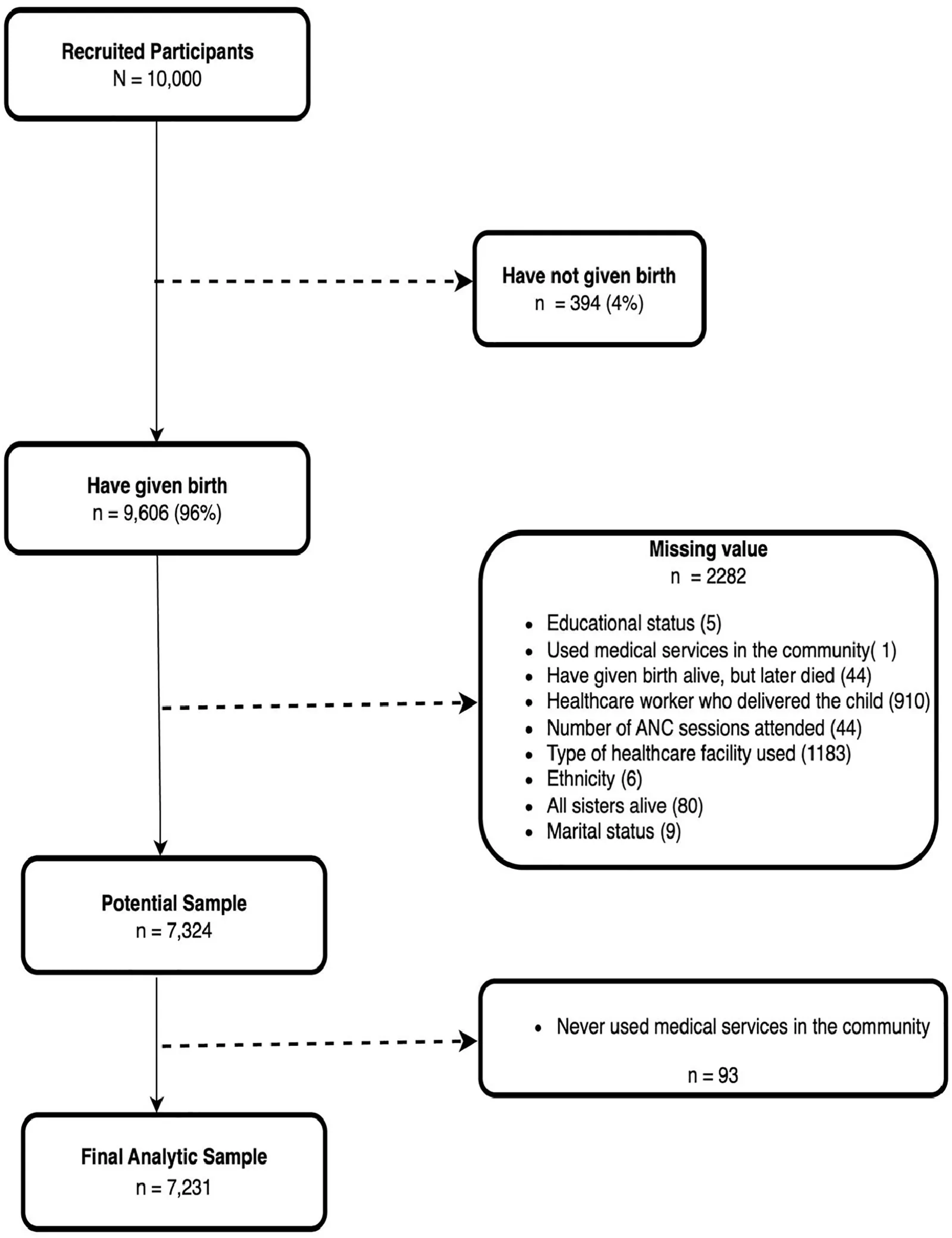
Analytic sample flow chart, 2022 survey of LARCHIS.

### Measures

#### Perceived quality of services and reproductive health-related variables.

Perceptions of care quality were assessed using a single item: *“How would you rate the quality of services you received?”* Responses were recorded on a 4-point Likert scale ranging from 1 (*poor*) to 4 (*excellent*). This measure is commonly used in population healthcare surveys; it does not depend on multiple dimensions of care quality, so it is not subjected to formal psychometric validation within this study

Reproductive health-related variables included infant loss, which was captured from the question “*Have you ever given birth to a child who was born alive but later died?*” and dichotomized as yes or no. Healthcare facility utilization was assessed by asking participants whether they had ever used maternal and child health services in their community, with responses dichotomized as yes or no. The number of antenatal care (ANC) visits during the most recent pregnancy was categorized into four groups in line with WHO guidelines on the number of antenatal care visits and a positive pregnancy experience: fewer than four visits, four or more visits, and “cannot remember” [25].

#### Covariates.

Participants’ age (years) and age at marriage (years) were measured continuously using self-reported age at the time of the survey and at first marriage, respectively. Ethnicity was self-reported and categorized as Yoruba, Hausa, Igbo, Non-Nigerian, or Others (which included all Nigerian ethnic groups not classified as Hausa, Igbo, or Yoruba). Educational attainment was self-reported and classified into three categories, namely, primary or less, secondary, or tertiary. Marital status was self-reported and re-categorized as married/living with partner, divorced/separated/widowed, or not married. Participants’ place of residence was recorded and collapsed from 20 local government areas (LGAs) into the recently established six healthcare districts, in accordance with the Lagos State Healthcare Service areas (Districts I-VI) [13].

### Analytic strategy

We examined the descriptive statistics for the overall sample and across the healthcare districts, using frequencies and percentages for categorical variables and means with standard deviations for continuous variables. We also conducted bivariable analyses to assess the relationships between care perception, health factors, and covariates using Pearson’s chi-squared test or Fisher’s exact test, as appropriate. Thirdly, we employed adjusted multinomial regression models to examine associations between perceived service quality and health care facility factors, adjusting for covariates. Finally, sensitivity and confirmatory analyses were performed using adjusted multivariable logistic mixed-effects models, with the healthcare district specified as a random effect. Adjusted odds ratios (aORs) with 95% confidence intervals (CIs) are reported. Statistical significance was assessed at p < 0.05. Analyses were conducted using R version 4.4.2 (R Core Team, R Foundation for Statistical Computing) [26].

### Data management

Data collection and management were conducted in real time using the Research Electronic Data Capture (REDCap) platform hosted at the Lagos State University College of Medicine (LASUCOM). To ensure accuracy and accountability, data collectors’ identifiers (name, initials, signature, and GPS location) were pre-programmed into the tablets and logged with each daily upload.

### Ethical considerations

The study was approved by the Lagos State University Teaching Hospital Ethics Committee (LREC/06/10/1789). Written informed consent was obtained from all participants, who were assured that their data would remain anonymous, be used solely for scientific purposes, and that they could withdraw at any time without penalty. The participants did not receive monetary or material incentives for completing the survey, and the study adhered to the Strengthening the Reporting of Observational Studies in Epidemiology (STROBE) guidelines [24].

## Results

### Sample characteristics by healthcare districts

As shown in Table 2 and Fig 1, 7,231 participants were included in the final analysis. Descriptive statistics for the overall sample and across health districts are presented in Table 2. The distribution of participants across health care districts was as follows: 1,191 (16.0%) from District I, 1,293 (18.0%) from District II, 1,073 (15%) from District III, 1,268 (17.0%) from District IV, 1,254 (17.0%) from District V, and 1,245 (17.0%) from District VI. The mean age was 31.2 years (SD = 5.9), and the mean age at marriage was 24.1 years (SD = 3.9); both were similar across districts (p > 0.05). Nearly all participants (96.0%) were married or cohabiting. Most women were Yoruba (67.7%), and educational attainment was generally high, with 85.9% reporting at least secondary education. Almost all participants (99.0%) reported using healthcare facilities in their communities. Regarding past pregnancies, 590 women (8.1%) reported infant loss. For prenatal care, 55.0% attended at least 4 sessions, 38.0% couldn’t recall how many visits they had, and 7.0% had 3 or fewer.

**Table 2:** Descriptive statistics of the participants’ characteristics stratified by Health district, 2022 Survey of LARCHIS.

Characteristic	Overall N = 7,324	Healthcare Districts					
		District 1N = 1,191	District 2N = 1,293	District 3N = 1,073	District 4N = 1,268	District 5N = 1,254	District 6N = 1,245
**Present Age**							
*Mean (SD)*	31.2 (5.9)	31.7 (5.9)	31.2 (6.1)	31.1 (5.7)	30.2 (6.2)	31.6 (5.5)	31.5 (5.7)
**Age at marriage**							
*Mean (SD)*	24.1 (3.9)	24.3 (4.0)	23.8 (4.2)	23.2 (4.0)	23.9 (3.7)	24.2 (3.8)	24.9 (3.9)
**Marital Status,** **No. (%)**							
*Married/Living with partner*	6,998.0	1,129.0 (16.1)	1,255.0 (17.9)	1,032.0 (14.7)	1,166.0 (16.7)	1,209.0 (17.3)	1,207.0 (17.2)
*Divorced/Separated/Widowed*	114.0	18.0 (15.8)	19.0 (16.7)	23.0 (20.2)	16.0 (14.0)	24.0 (21.1)	14.0 (12.3)
*Not Married*	212.0	44.0 (20.8)	19.0 (9.0)	18.0 (8.5)	86.0 (40.6)	21.0 (9.9)	24.0 (11.3)
**Education** attainment, **No. (%)**							
*<=Primary School*	1,035.0	170.0 (16.4)	176.0 (17.0)	137.0 (13.2)	199.0 (19.2)	233.0 (22.5)	120.0 (11.6)
*=Secondary School*	4,750.0	787.0 (16.6)	801.0 (16.9)	791.0 (16.7)	824.0 (17.3)	789.0 (16.6)	758.0 (16.0)
*>=College Degree*	1,539.0	234.0 (15.2)	316.0 (20.5)	145.0 (9.4)	245.0 (15.9)	232.0 (15.1)	367.0 (23.8)
**Ethnicity,** **No. (%)**							
*Hausa*	362.0	82.0 (22.7)	94.0 (26.0)	63.0 (17.4)	31.0 (8.6)	32.0 (8.8)	60.0 (16.6)
*Igbo*	1,388.0	169.0 (12.2)	191.0 (13.8)	148.0 (10.7)	192.0 (13.8)	358.0 (25.8)	330.0 (23.8)
*Non-Nigerian*	71.0	5.0 (7.0)	7.0 (9.9)	28.0 (39.4)	12.0 (16.9)	13.0 (18.3)	6.0 (8.5)
*Others*	545.0	66.0 (12.1)	108.0 (19.8)	84.0 (15.4)	39.0 (7.2)	168.0 (30.8)	80.0 (14.7)
*Yoruba*	4,958.0	869.0 (17.5)	893.0 (18.0)	750.0 (15.1)	994.0 (20.0)	683.0 (13.8)	769.0 (15.5)
**Ever Use a Health Facility,** **No. (%)**							
*No*	4.0	1.0 (25.0)	1.0 (25.0)	1.0 (25.0)	0.0 (0.0)	1.0 (25.0)	0.0 (0.0)
*Yes*	7,320.0	1,190.0 (16.3)	1,292.0 (17.7)	1,072.0 (14.6)	1,268.0 (17.3)	1,253.0 (17.1)	1,245.0 (17.0)
**Loss of Infant,** **No. (%)**							
*No*	6,734.0	1,074.0 (15.9)	1,148.0 (17.0)	999.0 (14.8)	1,206.0 (17.9)	1,158.0 (17.2)	1,149.0 (17.1)
*Yes*	590.0	117.0 (19.8)	145.0 (24.6)	74.0 (12.5)	62.0 (10.5)	96.0 (16.3)	96.0 (16.3)
**Number of ANC visit,** **No. (%)**							
*< 4 times*	344.0	32.0 (9.3)	45.0 (13.1)	91.0 (26.5)	119.0 (34.6)	44.0 (12.8)	13.0 (3.8)
*>=4 times*	4,145.0	554.0 (13.4)	757.0 (18.3)	538.0 (13.0)	784.0 (18.9)	712.0 (17.2)	800.0 (19.3)
*Cannot remember*	2,835.0	605.0 (21.3)	491.0 (17.3)	444.0 (15.7)	365.0 (12.9)	498.0 (17.6)	432.0 (15.2)
**All Sisters Alive,** **No. (%)**							
*No*	559.0	90.0 (16.1)	164.0 (29.3)	75.0 (13.4)	72.0 (12.9)	84.0 (15.0)	74.0 (13.2)
*Yes*	6,765.0	1,101.0 (16.3)	1,129.0 (16.7)	998.0 (14.8)	1,196.0 (17.7)	1,170.0 (17.3)	1,171.0 (17.3)

### Study population characteristics by perception of the quality of services

Perceptions of service quality varied significantly (p < 0.05) across health care districts, marital status, education, ethnicity, facility type, ANC attendance, and delay in arrival (>2 hours after labor onset) (all p < 0.001). District III had the highest proportion of women rating services as good (78.0%), while District I had the lowest (61.0%). Conversely, District I participants reported the highest proportion of those perceiving services as excellent (31.1%), whereas District III had the lowest (8.5%). By marital status, 72.0% of divorced/separated/widowed participants rated services as good, while 23.5% of married/cohabiting women rated services as excellent. Education showed a graded pattern: women with at most primary education most frequently rated services as good (70.7%), while tertiary-educated women rated services as excellent (28.1%). More non-Nigerians rated services as good (74.6%), whereas Yoruba and Igbo women had the highest proportion rating services as excellent (24.0%, each).

Among facility types, missionary/private centers had the highest proportion of excellent ratings (39.6%), while primary healthcare facilities were mostly perceived as good (70.0%). ANC attendance was also associated with women’s perceptions of quality: significantly more women reported having four or more ANC visits during pregnancy, and almost one quarter (24.4%) rated the services as excellent. Late arrival (>2 hours after labor onset) was associated with less favorable ratings. Facility-level characteristics, including waiting times, 24-hour availability/opening hours, doctor presence, and courteous staff, were significantly associated with higher service ratings, whereas barriers such as traffic, distance, cost, or spousal absence were not (Table 3).

**Table 3:** Bivariable associations between perceived quality of services, exposures, and covariates, 2022 Survey of LARCHIS.

**Healthcare district, No. (%)**					**<0.001**
*PHC District 1*	1,181.0	94.0 (8.0)	720.0 (61.0)	367.0 (31.1)	
*PHC District 2*	1,275.0	115.0 (9.0)	873.0 (68.5)	287.0 (22.5)	
*PHC District 3*	1,068.0	144.0 (13.5)	833.0 (78.0)	91.0 (8.5)	
*PHC District 4*	1,236.0	134.0 (10.8)	823.0 (66.6)	279.0 (22.6)	
*PHC District 5*	1,246.0	180.0 (14.4)	779.0 (62.5)	287.0 (23.0)	
*PHC District 6*	1,225.0	59.0 (4.8)	794.0 (64.8)	372.0 (30.4)	
**Marital** attainment**, No. (%)**					**0.004**
*Married/Living with partner*	6,914.0	679.0 (9.8)	4,609.0 (66.7)	1,626.0 (23.5)	
*Divorced/Separated/Widowed*	111.0	18.0 (16.2)	80.0 (72.1)	13.0 (11.7)	
*Not Married*	206.0	29.0 (14.1)	133.0 (64.6)	44.0 (21.4)	
**Level of education, No. (%)**					**<0.001**
*<=Primary School*	1,013.0	102.0 (10.1)	716.0 (70.7)	195.0 (19.2)	
*Secondary School*	4,689.0	456.0 (9.7)	3,175.0 (67.7)	1,058.0 (22.6)	
*Tertiary School*	1,529.0	168.0 (11.0)	931.0 (60.9)	430.0 (28.1)	
**Ethnicity, No. (%)**					**<0.001**
*Hausa*	358.0	31.0 (8.7)	245.0 (68.4)	82.0 (22.9)	
*Igbo*	1,386.0	173.0 (12.5)	875.0 (63.1)	338.0 (24.4)	
*Yoruba*	4,875.0	442.0 (9.1)	3,265.0 (67.0)	1,168.0 (24.0)	
*Non-Nigerian*	71.0	10.0 (14.1)	53.0 (74.6)	8.0 (11.3)	
*Other*	541.0	70.0 (12.9)	384.0 (71.0)	87.0 (16.1)	
**Facility type, No. (%)**					**<0.001**
*Missionary/Private healthcare center*	1,426.0	25.0 (1.8)	837.0 (58.7)	564.0 (39.6)	
*Primary healthcare center*	4,348.0	497.0 (11.4)	3,044.0 (70.0)	807.0 (18.6)	
*Secondary healthcare center*	1,377.0	199.0 (14.5)	886.0 (64.3)	292.0 (21.2)	
*Tertiary healthcare center*	80.0	5.0 (6.3)	55.0 (68.8)	20.0 (25.0)	
**Arrived more than 2 hours, No. (%)**					**<0.001**
*Do not know*	892.0	109.0 (12.2)	645.0 (72.3)	138.0 (15.5)	
*No*	6,021.0	576.0 (9.6)	3,965.0 (65.9)	1,480.0 (24.6)	
*Yes*	318.0	41.0 (12.9)	212.0 (66.7)	65.0 (20.4)	
**Number of ANC sessions attended, No. (%)**					**<0.001**
*< 4 times*	338.0	28.0 (8.3)	266.0 (78.7)	44.0 (13.0)	
*>=4 times*	4,101.0	374.0 (9.1)	2,728.0 (66.5)	999.0 (24.4)	
*Cannot remember*	2,792.0	324.0 (11.6)	1,828.0 (65.5)	640.0 (22.9)	
**Delay due to traffic, No. (%)**					0.071
*No*	7,083.0	703.0 (9.9)	4,727.0 (66.7)	1,653.0 (23.3)	
*Yes*	148.0	23.0 (15.5)	95.0 (64.2)	30.0 (20.3)	
**Delay due to husband absence, No. (%)**					0.6
*No*	7,156.0	716.0 (10.0)	4,772.0 (66.7)	1,668.0 (23.3)	
*Yes*	75.0	10.0 (13.3)	50.0 (66.7)	15.0 (20.0)	
**Delay due to far facility, No. (%)**					0.10
*No*	7,140.0	711.0 (10.0)	4,763.0 (66.7)	1,666.0 (23.3)	
*Yes*	91.0	15.0 (16.5)	59.0 (64.8)	17.0 (18.7)	
**Delay due to money, No. (%)**					0.8
*No*	7,215.0	724.0 (10.0)	4,812.0 (66.7)	1,679.0 (23.3)	
*Yes*	16.0	2.0 (12.5)	10.0 (62.5)	4.0 (25.0)	
**Delay due to other, No. (%)**					>0.9
*No*	7,162.0	718.0 (10.0)	4,777.0 (66.7)	1,667.0 (23.3)	
*Yes*	69.0	8.0 (11.6)	45.0 (65.2)	16.0 (23.2)	
**Short waiting time, No. (%)**					**<0.001**
*No*	4,191.0	589.0 (14.1)	2,891.0 (69.0)	711.0 (17.0)	
*Yes*	3,040.0	137.0 (4.5)	1,931.0 (63.5)	972.0 (32.0)	
**Long waiting time, No. (%)**					**<0.001**
*No*	6,485.0	371.0 (5.7)	4,461.0 (68.8)	1,653.0 (25.5)	
*Yes*	746.0	355.0 (47.6)	361.0 (48.4)	30.0 (4.0)	
**Facility opens 24 hours, No. (%)**					**<0.001**
*No*	2,516.0	507.0 (20.2)	1,791.0 (71.2)	218.0 (8.7)	
*Yes*	4,715.0	219.0 (4.6)	3,031.0 (64.3)	1,465.0 (31.1)	
**Staff are courteous, No. (%)**					**<0.001**
*No*	2,403.0	596.0 (24.8)	1,559.0 (64.9)	248.0 (10.3)	
*Yes*	4,828.0	130.0 (2.7)	3,263.0 (67.6)	1,435.0 (29.7)	
**Availability of doctors, No. (%)**					**<0.001**
*No*	2,951.0	524.0 (17.8)	2,017.0 (68.3)	410.0 (13.9)	
*Yes*	4,280.0	202.0 (4.7)	2,805.0 (65.5)	1,273.0 (29.7)	
**Skilled healthcare worker, No. (%)**					**<0.001**
*No*	1,995.0	481.0 (24.1)	1,252.0 (62.8)	262.0 (13.1)	
*Yes*	5,236.0	245.0 (4.7)	3,570.0 (68.2)	1,421.0 (27.1)	
**Facility often closed, No. (%)**					**<0.001**
*No*	7,091.0	669.0 (9.4)	4,773.0 (67.3)	1,649.0 (23.3)	
*Yes*	140.0	57.0 (40.7)	49.0 (35.0)	34.0 (24.3)	
**Rude staff, No. (%)**					**<0.001**
*No*	6,701.0	355.0 (5.3)	4,672.0 (69.7)	1,674.0 (25.0)	
*Yes*	530.0	371.0 (70.0)	150.0 (28.3)	9.0 (1.7)	
**Non-response of inquiries, No. (%)**					**<0.001**
*No*	6,940.0	504.0 (7.3)	4,761.0 (68.6)	1,675.0 (24.1)	
*Yes*	291.0	222.0 (76.3)	61.0 (21.0)	8.0 (2.7)	

^1^Pearson’s Chi-squared test; Fisher’s exact test

### Multivariable multinomial model between perceived quality of services, exposures, and covariates

#### Healthcare district.

Compared with District I, after adjusting for other predictors, participants in District III had significantly lower odds of rating services as good (aOR = 0.37; 95% CI: 0.24–0.56) or excellent (aOR = 0.08; 95% CI: 0.05–0.14) than rating them as poor/fair. Participants in District 5 were more likely to rate services as good (aOR = 1.77; 95% CI: 1.19–2.64) or excellent (aOR = 2.16; 95% CI: 1.39–3.37) than rating them as poor/fair. No significant differences were observed for Districts II, IV, or VI compared with District I (Table 4).

**Table 4:** Multinomial model between perceived quality of services, exposures, and covariates, 2022 Survey of LARCHIS.

Outcome	Characteristic	aOR	95% CI	p-value
Good	Healthcare service district			
	PHC District 1	—	—	
	PHC District 2	1.15	0.76, 1.75	0.5
	PHC District 3	0.37	0.24, 0.56	<0.001
	PHC District 4	0.84	0.55, 1.29	0.4
	PHC District 5	1.77	1.19, 2.64	0.005
	PHC District 6	1.54	0.93, 2.57	0.10
	Educational attainment			
	<=Primary School	—	—	
	Secondary School	0.91	0.65, 1.27	0.6
	Tertiary School	0.60	0.41, 0.89	0.010
	Marital status			
	Married/Living with partner	—	—	
	Divorced/Separated/Widowed	0.69	0.33, 1.46	0.3
	Not Married	0.87	0.47, 1.61	0.7
	Type of healthcare facility			
	Missionary/Private healthcare center	—	—	
	Primary healthcare center	0.64	0.40, 1.04	0.070
	Secondary healthcare center	0.45	0.27, 0.75	0.002
	Tertiary healthcare center	0.88	0.18, 4.17	0.9
	Ethnicity			
	Hausa	—	—	
	Igbo	0.88	0.49, 1.58	0.7
	Yoruba	1.15	0.67, 1.99	0.6
	Non-Nigerian	1.69	0.50, 5.67	0.4
	Other	0.68	0.36, 1.29	0.2
	Arrived more than 2 hours after labour started			
	Do not know	—	—	
	No	1.19	0.87, 1.64	0.3
	Yes	2.18	0.45, 10.5	0.3
	Number of ANC sessions attended			
	< 4 times	—	—	
	>=4 times	0.42	0.23, 0.74	0.003
	Cannot remember	0.33	0.18, 0.60	<0.001
	Delay due to traffic			
	No	—	—	
	Yes	0.51	0.13, 2.07	0.3
	Delay due to husband absence			
	No	—	—	
	Yes	0.57	0.13, 2.47	0.5
	Delay due to far facility			
	No	—	—	
	Yes	0.53	0.16, 1.68	0.3
	Delay due to money			
	No	—	—	
	Yes	2.97	0.05, 176	0.6
	Delay due to other			
	No	—	—	
	Yes	0.81	0.13, 5.00	0.8
	Short waiting time			
	No	—	—	
	Yes	0.74	0.57, 0.97	0.032
	Long waiting time			
	No	—	—	
	Yes	0.17	0.13, 0.23	<0.001
	Facility opens 24 hours			
	No	—	—	
	Yes	3.00	2.34, 3.84	<0.001
	Staff are courteous			
	No	—	—	
	Yes	3.34	2.59, 4.30	<0.001
	Doctors are always available			
	No	—	—	
	Yes	2.40	1.86, 3.10	<0.001
	Healthcare workers are skilled			
	No	—	—	
	Yes	3.19	2.51, 4.04	<0.001
	Facility often closed			
	No	—	—	
	Yes	0.41	0.21, 0.79	0.008
	Staff do not respond to inquiries			
	No	—	—	
	Yes	0.12	0.08, 0.18	<0.001
	Rude staff			
	No	—	—	
	Yes	0.15	0.11, 0.21	<0.001
Excellent	Healthcare service district			
	PHC District 1	—	—	
	PHC District 2	0.96	0.60, 1.51	0.8
	PHC District 3	0.08	0.05, 0.14	<0.001
	PHC District 4	0.70	0.44, 1.11	0.13
	PHC District 5	2.16	1.39, 3.37	<0.001
	PHC District 6	1.36	0.79, 2.32	0.3
	Educational attainment			
	<=Primary School	—	—	
	Secondary School	1.05	0.72, 1.53	0.8
	Tertiary School	0.83	0.54, 1.29	0.4
	Marital status			
	Married/Living with partner	—	—	
	Divorced/Separated/Widowed	0.34	0.13, 0.87	0.025
	Not Married	0.72	0.36, 1.46	0.4
	Type of healthcare facility			
	Missionary/Private healthcare center	—	—	
	Primary healthcare center	0.46	0.28, 0.75	0.002
	Secondary healthcare center	0.34	0.20, 0.59	<0.001
	Tertiary healthcare center	0.58	0.11, 2.97	0.5
	Ethnicity			
	Hausa	—	—	
	Igbo	0.84	0.44, 1.61	0.6
	Yoruba	1.19	0.65, 2.19	0.6
	Non-Nigerian	0.94	0.22, 4.10	0.94
	Other	0.40	0.19, 0.83	0.014
	Arrived more than 2 hours after labour started			
	Do not know	—	—	
	No	1.49	1.02, 2.17	0.039
	Yes	1.72	0.29, 10.4	0.6
	Number of ANC sessions attended			
	< 4 times	—	—	
	>=4 times	0.64	0.33, 1.24	0.2
	Cannot remember	0.51	0.26, 1.01	0.054
	Delay due to traffic			
	No	—	—	
	Yes	0.70	0.14, 3.49	0.7
	Delay due to husband absence			
	No	—	—	
	Yes	0.70	0.13, 3.74	0.7
	Delay due to far facility			
	No	—	—	
	Yes	0.58	0.15, 2.32	0.4
	Delay due to money			
	No	—	—	
	Yes	4.00	0.06, 284	0.5
	Delay due to other			
	No	—	—	
	Yes	1.80	0.23, 14.0	0.6
	Short waiting time			
	No	—	—	
	Yes	0.94	0.70, 1.26	0.7
	Long waiting time			
	No	—	—	
	Yes	0.05	0.03, 0.07	<0.001
	Facility opens 24 hours			
	No	—	—	
	Yes	10.8	8.07, 14.4	<0.001
	Staff are courteous			
	No	—	—	
	Yes	6.71	5.00, 9.00	<0.001
	Doctors are always available			
	No	—	—	
	Yes	3.14	2.35, 4.20	<0.001
	Healthcare workers are skilled			
	No	—	—	
	Yes	4.38	3.29, 5.84	<0.001
	Facility often closed			
	No	—	—	
	Yes	0.74	0.33, 1.63	0.5
	Staff do not respond to inquiries			
	No	—	—	
	Yes	0.06	0.03, 0.16	<0.001
	Rude staff			
	No	—	—	
	Yes	0.06	0.03, 0.13	<0.001

Abbreviations: CI = Confidence Interval, aOR = Adjusted Odds Ratio

#### Educational attainment.

Women with tertiary education were 40% less likely to rate services as good (aOR = 0.60; 95% CI: 0.41–0.89) than those with at most primary education, but there was no difference in the likelihood of excellent ratings (aOR = 0.83; 95% CI: 0.54–1.29). Sensitivity analyses also confirm that women with tertiary education were less likely overall to rate services as good or excellent (aOR = 0.62; 95% CI: 0.42–0.92; Supplemental Table 4B).

#### ANC sessions attended.

Compared with women with fewer than four ANC visits, those with four or more visits were less likely to rate services as good (aOR = 0.42; 95% CI: 0.23–0.74); there was no difference in excellent ratings (aOR = 0.64; 95% CI: 0.33–1.24). Women who could not recall the number of visits were also less likely to rate services as good (aOR = 0.33; 95% CI: 0.18–0.60), but there was no significant difference in excellent ratings (aOR = 0.51; 95% CI: 0.26–1.01; Table 4). The same rating trends were observed in the mixed-effects model: at least 4 visits (aOR = 0.43; 95% CI: 0.24–0.77) and could not recall (aOR = 0.34; 95% CI: 0.19–0.62).

#### Waiting time.

Longer wait times were significantly and negatively associated with perceived service quality. Women who waited longer were 83% less likely to perceive the service as good (aOR = 0.17; 95% CI: 0.13–0.23) and 99.95% less likely to rate it as excellent (aOR = 0.05; 95% CI: 0.03–0.07) than to perceive it as fair or poor. These findings were also confirmed by the Mixed-Effects model (Supplemental Table 4B), which showed that participants who waited longer were 84% less likely to perceive the service as good or excellent (aOR = 0.16; 95% CI: 0.12–0.21). However, there was no difference in the perception of the service as excellent (aOR = 0.94; 95% CI: 0.70–1.26) among those who waited a short time.

#### Availability and skillfulness of healthcare workers.

The operation and staffing of healthcare facilities significantly shape women’s perceptions of service quality. Facilities operating 24 hours are more likely to receive a good rating (aOR = 3.00; 95% CI: 2.34–3.84) or an excellent rating (aOR = 10.8; 95% CI: 8.07–14.4). The availability of doctors would also improve perceptions, increasing the odds of a good or an excellent rating by 140% (aOR = 2.40; 95% CI: 1.86–3.10) or 214% (aOR = 3.14; 95% CI: 2.35–4.20), respectively. Mixed-effects models confirm these findings, showing that 24-hour operation, doctor availability, and skilled staff would increase the likelihood of good or excellent ratings, while often-closed facilities reduce it by 56% (aOR = 0.44; 95%CI:0.23–0.84) (Supplemental Table 4B).

#### Staff hospitality.

Staff behavior strongly shapes service perceptions (Table 4). Courteous staff seems to increase the odds of a good rating by 234% (aOR = 3.34; 95% CI: 2.59–4.30) or an excellent rating by 571% (aOR = 6.71; 95% CI: 5.00–9.00). In contrast, unresponsiveness or rudeness would significantly lower the likelihood of positive perceptions. Women are 82% less likely to perceive services as good (aOR = 0.12; 95% CI: 0.08–0.18), and 99.94% less likely to perceive them as excellent (aOR = 0.06; 95% CI: 0.03–0.16) when staff do not respond to their inquiries. The odds of perceiving services as good would decrease by 85% (aOR = 0.15; 95% CI: 0.11–0.21) or excellent by 99.94% (aOR = 0.06; 95% CI: 0.03–0.13) when staff are rude. The observed positive associations of courteousness (aOR = 3.59; 95% CI: 2.79–4.62) and the inverse relationship of rudeness (aOR = 0.15; 95% CI: 0.11–0.20) with perceptions of service quality were confirmed by the mixed-effects models (Supplemental Table 4B).

## Discussions

Perceived quality reflects women’s subjective evaluation of services received, while satisfaction is often influenced by expectations and experiences. Objective quality, on the other hand, refers to adherence to clinical standards and technical competence. This study focuses on perceived quality, which plays a critical role in shaping future care-seeking behavior and trust in the health system. In Lagos State, Nigeria, women’s perceptions of maternal healthcare quality were strongly related to both structural and interpersonal factors, including provider waiting time, staff courtesy and responsiveness, facility preparedness, and the presence of doctors. Facilities operating 24 hours and those with skilled staff and doctors were associated with higher odds that women would rate services as good or excellent and would increase their utilization of the services during antenatal, delivery, and postnatal care [32–34]. In contrast, frequent facility closures and long waiting times significantly reduced positive perceptions. Similarly, staff behavior had a pronounced effect: courteous and responsive staff increased favorable evaluations, whereas rudeness or unresponsiveness sharply decreased positive evaluations. A systematic review assessing the determinants of maternal care satisfaction in Nigeria revealed that most studies reported high levels of satisfaction, with 20 studies reporting rates of 80% or higher [15,32]. These findings underscore that both health system readiness and person-centered care are critical determinants of perceived quality.

Geographic disparities in quality perceptions were particularly pronounced, with significant differences across the healthcare districts. Participants in District III were less likely to rate services as good or excellent than those in District VI, while women in District V had higher odds of negative ratings (fair/poor). These findings may reflect that Districts III and V primarily comprise rural LGAs, and peri-urban slums rely heavily on primary and secondary government-owned healthcare facilities, with only a few well-established private hospitals. Facility types also affected perceptions, with missionary and private centers earning more excellent ratings than public primary and secondary facilities [35]. These higher ratings from the missionary and private sectors might also be related to simpler governance structures and greater accountability than those of public facilities. These higher ratings are similar to findings from a Kenyan study, in which 98.1% of clients at private maternity facilities were attended within 30 minutes, versus 87% at public facilities, and the mean satisfaction scores were 4.46 and 4.60 out of 5 for public and private maternal healthcare facilities, respectively [23]. Greater dissatisfaction with publicly provided maternal health services was also associated with staff conduct in an Indian study, with dissatisfaction rates of 8.12% in public facilities compared with 2.96% in private establishments [36].

In line with this finding, a cross-national ecological study found that higher facility delivery rates were negatively correlated with maternal and neonatal mortality, underscoring the importance of perceived quality alongside access in improving outcomes [37]. Participants from health districts I and VI had higher excellent ratings and fewer complaints (fair or poor ratings). Varying satisfaction ratings for maternal healthcare services across Lagos State’s health districts may also reflect differences in the number and category of healthcare facilities (primary, secondary, or tertiary) within districts, health system responsiveness, provider-patient interactions, programmatic interventions, and other community-related factors, such as resource distribution and other social determinants of health. Districts with better-resourced facilities, strong leadership, or exposure to targeted quality improvement initiatives are more likely to receive excellent ratings from service users. Additionally, the ratio of government-owned to private facilities within these districts may contribute to the noted variations.

Education, antenatal care (ANC) attendance, and prior use of healthcare facilities shaped perceptions in nuanced ways. While tertiary-educated women were less likely to rate services as good, they were more likely to view overall service quality as good or excellent, suggesting that more highly educated women may have higher expectations and be more critical of care deficiencies [19]. Antenatal care engagement showed similar nuanced patterns. Significantly more women who attend four or more ANC visits rate the services as excellent (Table 3), which aligns with another study reporting a strong link between satisfaction with care and having more than four antenatal contacts during pregnancy. In that study, 91.3% of women with more contacts were satisfied, compared to 8.7% with fewer (p < 0.001) [38]. An increased number of antenatal visits, especially beyond four, improves perceptions of maternity care quality by increasing health education, personalized attention, and trust in providers, which are key factors in satisfaction and service experience [25].

Prior use of a healthcare facility, however, was associated with higher perceived quality, suggesting that familiarity with services builds trust and shapes positive experiences. These observations align with a study that found maternal education, wealth, and ANC attendance were significant predictors of neonatal outcomes in Nigeria, reinforcing the role of repeated interactions and familiarity with the healthcare system in shaping perceptions [25]. Our findings also offer insight into other contexts related to the persistent maternal and neonatal mortality challenges in Lagos. Studies have emphasized that facility delivery alone does not guarantee improved outcomes without an enabling environment [27]. This suggests that interventions should reduce barriers to first-time access to build familiarity and trust [39]. Despite widespread facility availability, negative perceptions of service quality may discourage early facility use, thereby contributing to stagnant progress toward global targets such as Sustainable Development Goal (SDG) 3 [40]. Appropriate interventions, ranging from community to health facility levels, are critical, as nearly two-thirds of deliveries in Nigeria occur at home [41].

To improve quality, enhance the user experience, and improve maternal and child health outcomes in Lagos State’s health districts, it is necessary to implement equity-driven, user-centered strategies that include appropriate quality control and accountability measures and are tailored to local contexts. Although some districts have existing infrastructure and are located in urban areas, closing the gap across all six districts will require a joint effort to improve quality, promote consistent ANC, provide respectful and responsive maternity care, expand access to financing, and engage local communities [42,43].

### Strengths and limitations

This study exhibited notable strengths. We leveraged and analyzed a large sample of 7,324 from the 2022 Lagos State Reproductive and Child Health Indicator Survey (LARCHIS), conducted across 20 LGAs and all 6 newly created health districts in Lagos State, Nigeria, with at least 1,070 participants per district. The inclusion of both structural (e.g., 24-hour operation, doctor availability) and interpersonal (e.g., staff courtesy, responsiveness) determinants provides a comprehensive assessment of perceived service quality. Multivariable multinomial and mixed-effects models were employed to analyze the data, allowing adjustment for confounders and accounting for clustering at the health district level, thereby yielding more robust and better estimates of the effects.

Despite these strengths, some limitations remain. First, as a cross-sectional study, we caution for inferring causality. Second, responses to questions about perceived care quality were self-reported and primarily retrospective, potentially introducing recall bias regarding past maternal health care experiences, but childbirth experiences are typically salient and therefore may be less prone to significant recall decay. Third, the use of a single-item measure of perceived quality limits the ability to identify specific components of care that women use to evaluate care quality. Future studies should be designed to capture multifaceted dimensions of care quality, such as technical competence and interpersonal experience, using multi-item scales and qualitative approaches to capture granular dimensions of maternal health care and better identify actionable drivers of quality improvement from women’s perspectives. Finally, the survey covered many quality indicators; some factors, such as supply stockouts, emergency response, or provider workload, were not measured and may have affected patients’ perceptions.

However, the study’s findings offer actionable insights to improve maternal healthcare delivery, highlighting the critical roles of facility preparedness, skilled staffing, and person-centered care in shaping women’s experiences and service utilization. These findings are especially relevant in urban and peri-urban settings in low- and middle-income countries, where access to facilities does not necessarily translate into improved outcomes. Policy efforts should prioritize both structural improvements (e.g., staffing, facility readiness) and interpersonal aspects of care (e.g., respectful maternity care).

## Conclusion

Women’s perceptions of maternal healthcare in Lagos are shaped by structural and interpersonal factors. Facilities with 24-hour service, skilled staff, and available doctors were rated higher, while closures and long waits lowered ratings. Staff courtesy, responsiveness, and professionalism were key, highlighting the importance of person-centered care alongside system readiness. Policies that strengthen structural and interpersonal aspects are vital to improve women’s healthcare experiences, ensure equitable access, and reduce maternal and newborn morbidity and mortality in Lagos, Nigeria.
