# Supplementary material for: Quality of maternal health services in Lagos, Nigeria: Women’s perspectives across healthcare districts
Source: PLoS One. 2026 Nov 6;21(11):e0359548. doi: 10.1371/journal.pone.0359548 (PMC13743031; doi:10.1371/journal.pone.0359548)
Supplement: Supplementary file 1 [file pone.0359548.s002.pdf]

QUALITY OF MATERNAL HEALTH SERVICES IN LAGOS, NIGERIA: WOMEN'S PERSPECTIVES ACROSS HEALTHCARE DISTRICTS

This manuscript examines women's perceptions of the quality of maternal health services in Lagos, Nigeria, using data from the Lagos Reproductive and Child Health Indicator Survey (LARCHIS).

This study addresses maternal healthcare quality in a large urban African setting. It uses a large, population-based dataset. District-level comparisons incorporation strengthens policy relevance.

However, there are major gaps in the reporting of the findings of the research, as mentioned in the following comments and questions.

INTRODUCTION

The introduction needs to be well-arranged, addressing the following key points

- The specific deficiencies in existing literature should be identified by this study.
- Significance of the problem to the targeted respondent needs to be better developed.
- The research gap is not clearly explained. More explanation on the gap that this study is willing to fill will be appreciated.
- Kindly highlight the primary and secondary research questions to be addressed
- According to the topic, you are looking at "women's perceptions of the quality of maternal health services" which they have received, not how barriers to access and perception of quality shape maternal health-seeking behavior. Kindly clarify this statement made in line 118-120
- Explicit differentiation from previous analyses based on the Nigerian Demographic and Health Survey (NDHS) will be appreciated.
- What is being referred to as formal health care system, as stated in line 114?
  "Moreover, most existing research in Nigeria is derived from hospital-based studies, often at tertiary centers, which may not fully capture the realities of women who deliver outside **formal healthcare systems".**
- As stated in line 112 to 114, does LARCHIS capture the reality of women who delivered outside hospital system?
  "Moreover, most existing research in Nigeria is derived from hospital-based studies, often at tertiary centers, which may not fully capture the realities of women who deliver outside **formal healthcare systems** "
- Dear author, the sentence in lines 120 to 122 looks vague and difficult to interpret; consider rephrasing the sentence so as to understand the context. *"By situating maternal mortality within the paradox of Lagos, an urban megacity where abundant infrastructure coexists with poor maternal outcomes, this study seeks to identify structural and perceptual barriers driving healthcare decisions."*

STUDY DESIGN

- Provide detailed information on the weighting strategy and sampling methods.
- Inclusion criteria for the participants should be included.
- Line 137: Was it the time that the LARCHIS project was conducted that is being referred to here, or the date when your research was carried out? Kindly state explicitly when your research was done and when the LARCHIS data was collected as well.
- Line 148, kindly add to the statement that no **"new"** participants were enrolled.
- STROBES guideline should be added.
- Analytic sample flow charts not visible fig 1
  Under the "Ethics statement," NA was written: this should be corrected, even though no new participants were used but it still involves human participants. And the "NA" that was filled contradicts your statement in lines 209 to 212.

Statistical Analysis

- Model specification is insufficiently described.
- How were missing data managed
- Include a discussion of multicollinearity testing.
- Quality ratings categorized as Poor/Fair, Good, and Excellent. Was this a self-reported single-item measurement? Was the scale validated? Was any psychometric testing performed?

DISCUSSION

Clarify differences between satisfaction, perceived quality, and actual quality.
Avoid overstating links between perception and mortality reduction unless directly supported by their data.

DATA AVAILABILITY

- This manuscript states that "all relevant data are within the manuscript and its Supporting Information files". I will like to confirm "Is the full LARCHIS dataset publicly accessible?".
- If it is available, a repository link to the data set or an access protocol number should be provided
