# Supplementary material for: Quality of maternal health services in Lagos, Nigeria: Women’s perspectives across healthcare districts
Source: PLoS One. 2026 Nov 6;21(11):e0359548. doi: 10.1371/journal.pone.0359548 (PMC13743031; doi:10.1371/journal.pone.0359548)
Supplement: Supplementary file 2 [file pone.0359548.s001.docx]

**PLOS ONE**

**Quality of maternal health services in Lagos, Nigeria: Women’s perspectives across health care districts**

The study was designed to investigate the quality of maternal healthcare services in Lagos State of Nigeria from the perspectives of women themselves. The data was obtained from an existing dataset obtained in 2022 from a survey of reproductive and maternal and child health indicators in Lagos State. The study addresses an important challenge but has some limitations which may constrain the utility of the results for policy and practice. While the perceptive of women on quality of care was the main outcome variable, the quantitative design of the study has not allowed for deeper reflections and elicitations on the components of care quality. The responses simply categorized quality as poor, good, or excellent without identifying components that were “good” or “excellent” to enable follow-up remediating actions to be undertaken. I believe a an add-on qualitative design or a more specific design asking questions on specific components of maternal care would have enabled the women to identify factors that could be acted upon by policymakers to improve the quality of care in Lagos State. However, I believe this point can be addressed as a limitation to the study in the discussion section of the paper.

Additionally, the study date being 2022, the timing of previous maternity visits is unclear, making recall bias a real threat to the internal validity of the study.

I have few additional comments as follows:

*Novelty* – There have been several publications on quality of maternal care in many parts of Nigeria, as well as other parts of Africa. The paper would do better if these past studies are summarized in a way to enable the identification of the specific novelty and research gaps being addressed by this study.

*Internal validity* – The study uses an existing dataset from the 2022 – the Lagos Reproductive and Child Health Indicator Survey. More details about this dataset, including the data collection process and the quality, should be provided to enable readers see how the study guide/protocol/questionnaire align with the specific objectives of this study.

*External validity* – The limitations of the study and the implications of the results for policy and practice should be better discussed in the discussion section

*Grammatical flow and understanding* – the manuscript reads well with good flow of ideas. However, there are a few typos that need to be addressed before the paper can be accepted.

My overall recommendation is for the authors to revise and resubmit.
