# Supplementary material for: Quality of maternal health services in Lagos, Nigeria: Women’s perspectives across healthcare districts
Source: PLoS One. 2026 Nov 6;21(11):e0359548. doi: 10.1371/journal.pone.0359548 (PMC13743031; doi:10.1371/journal.pone.0359548)
Supplement: Supplementary file 3 [file pone.0359548.s003.docx]

**MANUSCRIPT #:** PLOS ONE [PONE-D-25-59599]

**Title:** Quality of Maternal Health Services in Lagos, Nigeria: Women's Perspectives Across Healthcare Districts

**Response to the Editor and Reviewers**

We sincerely thank the Editor and Reviewers for their careful, thoughtful, and constructive evaluation of our manuscript, “Quality of Maternal Health Services in Lagos, Nigeria: Women’s Perspectives Across Healthcare Districts.” We are grateful for the opportunity to revise the manuscript and have carefully considered all comments. The feedback has substantially strengthened the rigor, clarity, and interpretability of our work. We have revised the manuscript to align closely with PLOS ONE editorial standards. Below, we provide detailed responses to each reviewer's comments.

**Summarization of Edits from Reviews and Author Responses.**

**Reviewer #1**

**Comment #1**: The quantitative design limits deeper insights into specific components of care quality. The responses simply categorized quality as poor, good, or excellent without identifying
components that were “good” or “excellent” to enable follow-up remediating actions to be
undertaken. I believe an add-on qualitative design or a more specific design, asking questions
on specific components of maternal care would have enabled the women to identify factors
that could be acted upon by policymakers to improve the quality of care in Lagos State.
However, I believe this point can be addressed as a limitation of the study in the discussion
section of the paper.

**Response**: We have expanded the Limitation section to explicitly acknowledge this limitation. We now emphasize that the LARCHIS dataset was not designed to capture granular dimensions of care quality, such as technical competence or interpersonal experience, and we highlight the need for future studies to use multi-item scale constructs to capture granular dimensions of maternal health care, mixed-methods studies, including qualitative approaches, to better identify actionable drivers of quality improvement from women’s perspectives. (**Page 28**).

**Comment #2**: The study date being 2022, the timing of previous maternity visits is unclear, making recall bias a real threat to the internal validity of the study.

**Response:** We have clarified in the Methods section that the LARCHIS survey was conducted in 2022 and collected retrospective data on recent maternal health experiences. We have also expanded the Discussion to explicitly acknowledge recall bias as a limitation, noting that while subjective perceptions may be affected, childbirth experiences are typically salient and therefore may be less prone to significant recall decay (**page 28**).

**Comment #3**: Novelty – There have been several publications on quality of maternal care in many parts of Nigeria, as well as other parts of Africa. The paper would do better if these past studies are summarized in a way to enable the identification of the specific novelty and research gaps being addressed by this study.

**Response:** We have substantially strengthened the Introduction by synthesizing the existing literature on maternal healthcare quality in Nigeria and sub-Saharan Africa. We now more clearly identify gaps in prior studies, including their reliance on facility-based or tertiary hospital data, limited use of population-based datasets, and lack of sub-state analyses. We also explicitly articulate the novelty of our study, particularly its use of a large, population-based dataset and its focus on district-level variation in women’s perceived quality of care. (**page 7**).

**Comment # 4**: Internal validity – The study uses an existing dataset from the 2022 – the Lagos Reproductive and Child Health Indicator Survey. More details about this dataset, including the data collection process and the quality, should be provided to enable readers see how the study
guide/protocol/questionnaire align with the specific objectives of this study.

**Response:** We have expanded the Methods section to provide a clearer description of the LARCHIS survey, including its sampling design, data collection procedures, and relevance to our research objectives. While we continue to cite the primary LARCHIS publication, we have ensured that sufficient detail is now included within the manuscript to allow readers to assess data quality and alignment with study aims. (**page 8**).

**Comment #5**: External validity – The limitations of the study and the implications of the results for policy and practice should be better discussed in the discussion section

**Response:** We have strengthened the Discussion to better address issues of external validity and policy relevance. We now discuss more clearly the generalizability of the findings to similar urban contexts and provide more detailed implications for policy and practice, particularly for improving person-centered care and addressing district-level disparities. We have also carefully reviewed the manuscript for typographical and grammatical errors and corrected these throughout.

**Reviewer #2**

We thank Reviewer #2 for their detailed and constructive feedback.

**Comment #1**: - The specific deficiencies in existing literature should be identified by this study. The significance of the problem to the targeted respondent needs to be better developed. The research gap is not clearly explained. More explanation on the gap that this study is willing to fill will be appreciated, and kindly highlight the primary and secondary research questions to be addressed

Response: We have substantially reorganized sections of the Introduction to improve clarity and logical flow. The revised Introduction now more clearly outlines deficiencies in the existing literature, strengthens the articulation of the public health significance of maternal healthcare quality in Lagos, and explicitly defines the research gap this study addresses. We have also added clear statements of the primary and secondary research objectives to guide the reader. (**page 6**).

**Comment #2**: According to the topic, you are looking at “women's perceptions of the quality of maternal health services” which they have received, not how barriers to access and perception of quality shape maternal health-seeking behavior. Kindly clarify this statement made in lines 118-120

**Response:** We have revised the relevant section to ensure that the manuscript consistently reflects that the study focuses on women’s perceptions of the quality of care received, rather than determinants of care-seeking behavior. Any references to health-seeking decisions are now framed as contextual background rather than study outcomes. (**pages 6-7**)

**Comment #3**: Explicit differentiation from previous analyses based on the Nigerian Demographic and Health Survey (NDHS) will be appreciated.

**Response:** We have added text explicitly noting in the Method section that our study is a secondary analysis of the LACHIS and NDHS datasets, which do not capture state-level granularity or specific factors such as newly created health districts in Lagos, as offered by LARCHIS. This distinction highlights the added value of our analysis in understanding district-level variation within Lagos. (**page 8**).

**Comment #4**: What is being referred to as formal health care system, as stated in line 114? As stated in line 112 to 114, does LARCHIS capture the reality of women who delivered outside hospital system? “Moreover, most existing research in Nigeria is derived from hospital-based studies, often at tertiary centers, which may not fully capture the realities of women who deliver outside formal healthcare systems”.

**Response:** We have clarified that “formal healthcare system” refers to recognized public and private health facilities staffed by trained providers, including primary, secondary, and tertiary institutions. We have also addressed the question of whether LARCHIS captures women delivering outside hospital settings by clarifying that while the dataset includes a range of facility types, it may underrepresent women who deliver entirely outside the formal healthcare system, which is now acknowledged as a limitation. (**page 6**)

**Comment #5**: - Dear author, the sentence in lines 120 to 122 looks vague and difficult to interpret; consider rephrasing the sentence so as to understand the context. “By situating maternal mortality within the paradox of Lagos, an urban megacity where abundant infrastructure coexists with poor maternal outcomes, this study seeks to identify structural and perceptual barriers driving healthcare decisions.”

**Response:** The previously unclear sentence regarding the “paradox of Lagos” has been rewritten to improve clarity and readability.

**Comment #6**: Provide detailed information on the weighting strategy and sampling methods. Inclusion criteria for the participants should be included. Line 137: Was it the time that the LARCHIS project was conducted that is being referred to here, or the date when your research was carried out? Kindly state explicitly when your research was done and when the LARCHIS data was collected as well.

**Response:** We have expanded the Methods section to provide detailed information on sampling procedures, weighting strategies, and inclusion criteria. We have clarified the data collection timeline (April-August 2022). (**pages 8 and 9**).

**Comment #7**: - Line 148, kindly add to the statement that no “new” participants were enrolled.

Response: We have stated in the manuscript that no new participants were enrolled in this secondary analysis. (**page 9**)

**Comment #8**: - STROBES guideline should be added. Analytic sample flow charts not visible in Figure 1

**Response:** We have added a statement confirming adherence to STROBE guidelines and included the checklist as supplementary material. The analytic sample flow chart (Figure 1) has been corrected to ensure visibility. (**Page 10**)

**Comment #9**: Under the “Ethics statement,” NA was written: this should be corrected, even though no new participants were used but it still involves human participants. And the “NA” that was filled contradicts your statement in lines 209 to 212.

**Response**: We appreciate the Reviewer’s attention to the inconsistency in the ethics statement. This has been fully corrected, with removal of “N/A” and inclusion of the ethics approval body, approval number, consent procedures, and data anonymization details.

**Comment #10**: - Model specification is insufficiently described.

Response: The Statistical Analysis section has been expanded to provide a more detailed description of model specification, including the use of multinomial regression and logistic mixed-effects modeling with the healthcare district specified as a random effect. (**page 12**)

**Comment #11**: - How were missing data managed

Response: We have added detailed information on how we handled missing data and how we obtained a final complete-case sample of 7,324 records, with at least 1,070 records per health district, which is large for the study. (**Page 9 and Figure 1 on page 10**)

**Comment #12**: - Include a discussion of multicollinearity testing.

**Response:** Below is the test for multicollinearity; no concerns were identified, as each GVI^(1/(2*Df)) is less than 2.

**Comment #13**: - Quality ratings categorized as Poor/Fair, Good, and Excellent. Was this a self-reported single-item measurement? Was the scale validated? Was any psychometric testing performed?

**Response:** The rating scale is a standard, widely validated single-item measure commonly used in population health studies, particularly in patient experience surveys to capture subjective evaluations of care. Usually, the scale is as follows: 1-Poor, 2-Fair, 3-Good, 4-Very Good, 5-Excellent. More details on the scale's validation can be found in the following studies: "Validating Patient Perspectives: A Study on the Reliability of Satisfaction Survey Tools" (<https://pmc.ncbi.nlm.nih.gov/articles/PMC11881768/>) and "Routine measurement of patient experience" (<https://pmc.ncbi.nlm.nih.gov/articles/PMC9884896/>). It does not require a validated psychometric scale because it is not a multi-item scale. However, we include it in the limitations and suggest that future studies use a multi-item scale to assess the granularity of maternal health care visit experiences.

**Comment #14**: Clarify differences between satisfaction, perceived quality, and actual quality.
Avoid overstating links between perception and mortality reduction unless directly supported by their data.

**Response:** We have clarified that the outcome variable was a self-reported single-item measure of perceived quality, a standard and commonly used public health survey item. In addition, we have revised the Discussion to clearly distinguish between perceived quality, patient satisfaction, and objective measures of care quality, and we have avoided overstating any links to maternal health outcomes.

**Comment # 15:** Is the full LARCHIS dataset publicly accessible? If it is available, a repository link to the data set or an access protocol number should be provided

**Response:** We have revised the Data Availability Statement to clarify that LARCHIS data are not fully publicly accessible and have provided a clear description of the data access process in accordance with PLOS ONE policy.

**Reviewer #3**

We thank Reviewer #3 for recognizing the scientific and public health value of this work and for the constructive suggestions provided. We also provide detailed responses to each comment below.

**Comment #1**: Ethics Statement – Clarification Required.

**Response:** We have corrected all inconsistencies by clearly stating the approving ethics committee, providing the approval number, and describing consent and anonymization procedures. All instances of “N/A” have been removed.

**Comment #2**: Data Availability Statement – Needs Precision

**Response:** We have also revised the Data Availability Statement to clearly specify data ownership and access procedures, ensuring alignment with PLOS ONE requirements. To strengthen the Abstract, we have included a key quantitative finding to enhance the impact and clarity of the study’s results.

**Comment #3:** Abstract - Consider adding one quantitative result (e.g., strongest adjusted odds ratio) to strengthen impact.

**Response:** We have revised the abstract by adding some quantitative results. (**Page 3**)

**Comment #4**: Clarify Causal Language

**Response:** We have carefully reviewed the manuscript and replaced causal terminology with appropriate language describing observational associations throughout. We have also added an explicit statement in the Discussion acknowledging that causal inferences cannot be drawn because of the cross-sectional nature of the study.

**Comment #5:** Additional suggested enhancement for discussions and interpretation: Expand briefly on why missionary/private facilities may outperform public ones beyond staffing (e.g., governance, accountability) and provide additional practical implications for Lagos State health system reform.

**Response:** We expanded the Discussion to elaborate on potential reasons why private and missionary facilities may outperform public facilities, including differences in governance, accountability, and patient-centered care practices. We have also strengthened the policy implications for health system reform in Lagos State. (**Page 26**)

**Reviewer #4**

We thank Reviewer #4 for their positive assessment of the study’s scientific rigor and relevance. We also provide detailed responses to each comment below

**Comment #1**: I said no because the dataset of the current manuscript is not findable to my search. however, if the PLOS editors are aware of this i will be convinced to change my answer to "YES"

**Response:** We have clarified the dataset's status and revised the Data Availability Statement to align with PLOS ONE policies.

**Comment #2**: Of the 44 participants excluded from the analysis of 10,000, one exclusion criterion was reporting a live birth that subsequently resulted in infant death. Nevertheless, the questionnaire included a reproductive health variable assessing infant loss, measured by the question: “Have you ever given birth to a child who was born alive but later died?” Based on this variable, 590 women reported experiencing infant loss. If the exclusion was actually due to missing/inconsistent responses, that should be clarified, because otherwise it looks contradictory.

**Response:** We have clarified that exclusions were due to missing or inconsistent responses rather than infant death per se, and we have revised the text to eliminate any ambiguity (**see Figure 1**).

**Comment #3**: In the data management section, what has been mentioned is how the primary data was managed. The dataset was screened for completeness and consistency, and the predefined inclusion and exclusion criteria were applied. However, additional data management steps undertaken for the current study shall be described.

**Response:** We have expanded the Data Management section to describe additional steps undertaken in this analysis, including data cleaning, variable recoding, consistency checks, and handling of missing data. This provides greater transparency regarding how the dataset was prepared for analysis. (**Page 10**)

**Closing Statement**

We again thank the Editor and Reviewers for their valuable feedback. The revisions have significantly improved the clarity, methodological rigor, and overall quality of the manuscript. We believe the manuscript is now substantially strengthened and meets the standards required for publication in PLOS ONE. We appreciate your consideration and look forward to your response.
