# Supplementary material for: Quality of maternal health services in Lagos, Nigeria: Women’s perspectives across healthcare districts
Source: PLoS One. 2026 Nov 6;21(11):e0359548. doi: 10.1371/journal.pone.0359548 (PMC13743031; doi:10.1371/journal.pone.0359548)
Supplement: Supplementary file 4 [file pone.0359548.s004.docx]

**MANUSCRIPT #:** PLOS ONE [PONE-D-25-59599]

**Title:** Quality of Maternal Health Services in Lagos, Nigeria: Women's Perspectives Across Healthcare Districts

**Response to the Editor and Reviewers**

We sincerely thank the Editor and Reviewers for their careful, thoughtful, and constructive evaluation of our manuscript, “Quality of Maternal Health Services in Lagos, Nigeria: Women’s Perspectives Across Healthcare Districts.” We have carefully considered the comments and have addressed the requested revisions. Below, we provide our response to each comment and indicate the corresponding changes made to the manuscript.

**Summary of Authors’ Responses.**

**Academic Editor**

**Comment #1**: Kindly revise the Abstract section to ensure that the word counts do not exceed 300 words.

**Response**: We have revised the Abstract to comply with the PLOS ONE requirement of no more than 300 words. The total word count of the abstract is now 269 words.

**Comment #2**: Please ensure that you refer to Table 2 in your text, as, if accepted, production will need this reference to link the reader to the Table.

**Response:** Table 2 is now referenced in the manuscript. (**page 13**).
