# Supplementary material for: Quality of maternal health services in Lagos, Nigeria: Women’s perspectives across healthcare districts
Source: PLoS One. 2026 Nov 6;21(11):e0359548. doi: 10.1371/journal.pone.0359548 (PMC13743031; doi:10.1371/journal.pone.0359548)
Supplement: Peer review history

# Peer review: Decision Letter 0

**DOI:** 10.1371/journal.pone.0359548.r001

**Authors:** Ayodeji Babatunde Oginni

22 Mar 2026

Dear Dr.  Momodu,

plosone@plos.org. When you're ready to submit your revision, log on to https://www.editorialmanager.com/pone/ and select the 'Submissions Needing Revision' folder to locate your manuscript file.

- A letter that responds to each point raised by the academic editor and reviewer(s). You should upload this letter as a separate file labeled 'Response to Reviewers'.
- A marked-up copy of your manuscript that highlights changes made to the original version. You should upload this as a separate file labeled 'Revised Manuscript with Track Changes'.
- An unmarked version of your revised paper without tracked changes. You should upload this as a separate file labeled 'Manuscript'.

If you would like to make changes to your financial disclosure, please include your updated statement in your cover letter. Guidelines for resubmitting your figure files are available below the reviewer comments at the end of this letter.

If applicable, we recommend that you deposit your laboratory protocols in protocols.io to enhance the reproducibility of your results. Protocols.io assigns your protocol its own identifier (DOI) so that it can be cited independently in the future. For instructions see: https://journals.plos.org/plosone/s/submission-guidelines#loc-laboratory-protocols. Additionally, PLOS ONE offers an option for publishing peer-reviewed Lab Protocol articles, which describe protocols hosted on protocols.io. Read more information on sharing protocols at https://plos.org/protocols?utm_medium=editorial-email&utm_source=authorletters&utm_campaign=protocols.

We look forward to receiving your revised manuscript.

Kind regards,

Ayodeji Babatunde Oginni

Academic Editor

PLOS One

Journal Requirements:

When submitting your revision, we need you to address these additional requirements.

1.Please ensure that your manuscript meets PLOS ONE's style requirements, including those for file naming. The PLOS ONE style templates can be found at

https://journals.plos.org/plosone/s/file?id=wjVg/PLOSOne_formatting_sample_main_body.pdf and

https://journals.plos.org/plosone/s/file?id=ba62/PLOSOne_formatting_sample_title_authors_affiliations.pdf

2. Please include a complete copy of PLOS’ questionnaire on inclusivity in global research in your revised manuscript. Our policy for research in this area aims to improve transparency in the reporting of research performed outside of researchers’ own country or community. The policy applies to researchers who have travelled to a different country to conduct research, research with Indigenous populations or their lands, and research on cultural artefacts. The questionnaire can also be requested at the journal’s discretion for any other submissions, even if these conditions are not met. Please find more information on the policy and a link to download a blank copy of the questionnaire here: https://journals.plos.org/plosone/s/best-practices-in-research-reporting. Please upload a completed version of your questionnaire as Supporting Information when you resubmit your manuscript.

3. We notice that your supplementary figures are uploaded with the file type 'Figure'. Please amend the file type to 'Supporting Information'. Please ensure that each Supporting Information file has a legend listed in the manuscript after the references list.

4. We notice that your supplementary figures are uploaded with the file type 'Figure'. Please amend the file type to 'Supporting Information'. Please ensure that each Supporting Information file has a legend listed in the manuscript after the references list.

5. If the reviewer comments include a recommendation to cite specific previously published works, please review and evaluate these publications to determine whether they are relevant and should be cited. There is no requirement to cite these works unless the editor has indicated otherwise.

Additional Editor Comments:

**I recommend that you thoroughly consider and respond to all reviewers' comments to improve the manuscript's quality. From my evaluation of the manuscript, I can see that you used Multinomial Logistic Regression, and I know that when you exponentiate the model's coefficients, you don’t get odds ratios; you get Relative Risk Ratios. So, why did you present odds ratios in your Table 4?**

**Comments to the Author**

1. Is the manuscript technically sound, and do the data support the conclusions?

Reviewer #1: Partly

Reviewer #2: Partly

Reviewer #3: Yes

Reviewer #4: Yes

**********

2. Has the statistical analysis been performed appropriately and rigorously?

Reviewer #1: Yes

Reviewer #2: No

Reviewer #3: Yes

Reviewer #4: Yes

**********

3. Have the authors made all data underlying the findings in their manuscript fully available?

The PLOS Data policy

Reviewer #1: Yes

Reviewer #2: No

Reviewer #3: No

Reviewer #4: No

**********

4. Is the manuscript presented in an intelligible fashion and written in standard English?

Reviewer #1: Yes

Reviewer #2: Yes

Reviewer #3: Yes

Reviewer #4: Yes

**********

Reviewer #1: Quality of maternal health services in Lagos, Nigeria: Women’s perspectives across health

care districts

The study was designed to investigate the quality of maternal healthcare services in Lagos State

of Nigeria from the perspectives of women themselves. The data was obtained from an existing

dataset obtained in 2022 from a survey of reproductive and maternal and child health

indicators in Lagos State. The study addresses an important challenge but has some limitations

which may constrain the utility of the results for policy and practice. While the perceptive of

women on quality of care was the main outcome variable, the quantitative design of the study

has not allowed for deeper reflections and elicitations on the components of care quality. The

responses simply categorized quality as poor, good, or excellent without identifying

components that were “good” or “excellent” to enable follow-up remediating actions to be

undertaken. I believe a an add-on qualitative design or a more specific design asking questions

on specific components of maternal care would have enabled the women to identify factors

that could be acted upon by policymakers to improve the quality of care in Lagos State.

However, I believe this point can be addressed as a limitation to the study in the discussion

section of the paper.

Additionally, the study date being 2022, the timing of previous maternity visits is unclear,

making recall bias a real threat to the internal validity of the study.

I have few additional comments as follows:

Novelty – There have been several publications on quality of maternal care in many parts of

Nigeria, as well as other parts of Africa. The paper would do better if these past studies are

summarized in a way to enable the identification of the specific novelty and research gaps

being addressed by this study.

Internal validity – The study uses an existing dataset from the 2022 – the Lagos Reproductive

and Child Health Indicator Survey. More details about this dataset, including the data collection

process and the quality, should be provided to enable readers see how the study

guide/protocol/questionnaire align with the specific objectives of this study.

External validity – The limitations of the study and the implications of the results for policy and

practice should be better discussed in the discussion section

Grammatical flow and understanding – the manuscript reads well with good flow of ideas.

However, there are a few typos that need to be addressed before the paper can be accepted.

My overall recommendation is for the authors to revise and resubmit.

Reviewer #2: QUALITY OF MATERNAL HEALTH SERVICES IN LAGOS, NIGERIA: WOMEN’S PERSPECTIVES ACROSS HEALTHCARE DISTRICTS

This manuscript examines women's perceptions of the quality of maternal health services in Lagos, Nigeria, using data from the Lagos Reproductive and Child Health Indicator Survey (LARCHIS).

This study addresses maternal healthcare quality in a large urban African setting. It uses a large, population-based dataset. District-level comparisons incorporation strengthens policy relevance.

However, there are major gaps in the reporting of the findings of the research, as mentioned in the following comments and questions.

INTRODUCTION

The introduction needs to be well-arranged, addressing the following key points

- The specific deficiencies in existing literature should be identified by this study.

- Significance of the problem to the targeted respondent needs to be better developed.

- The research gap is not clearly explained. More explanation on the gap that this study is willing to fill will be appreciated.

- Kindly highlight the primary and secondary research questions to be addressed

- According to the topic, you are looking at “women's perceptions of the quality of maternal health services” which they have received, not how barriers to access and perception of quality shape maternal health-seeking behavior. Kindly clarify this statement made in line 118-120

- Explicit differentiation from previous analyses based on the Nigerian Demographic and Health Survey (NDHS) will be appreciated.

- What is being referred to as formal health care system, as stated in line 114?

“Moreover, most existing research in Nigeria is derived from hospital-based studies, often at tertiary centers, which may not fully capture the realities of women who deliver outside formal healthcare systems”.

- As stated in line 112 to 114, does LARCHIS capture the reality of women who delivered outside hospital system?

“Moreover, most existing research in Nigeria is derived from hospital-based studies, often at tertiary centers, which may not fully capture the realities of women who deliver outside formal healthcare systems “

- Dear author, the sentence in lines 120 to 122 looks vague and difficult to interpret; consider rephrasing the sentence so as to understand the context.“By situating maternal mortality within the paradox of Lagos, an urban megacity where abundant infrastructure coexists with poor maternal outcomes, this study seeks to identify structural and perceptual barriers driving healthcare decisions.”

STUDY DESIGN

- Provide detailed information on the weighting strategy and sampling methods.

- Inclusion criteria for the participants should be included.

- Line 137: Was it the time that the LARCHIS project was conducted that is being referred to here, or the date when your research was carried out? Kindly state explicitly when your research was done and when the LARCHIS data was collected as well.

- Line 148, kindly add to the statement that no “new” participants were enrolled.

- STROBES guideline should be added.

- Analytic sample flow charts not visible fig 1

Under the “Ethics statement,” NA was written: this should be corrected, even though no new participants were used but it still involves human participants. And the “NA” that was filled contradicts your statement in lines 209 to 212.

Statistical Analysis

- Model specification is insufficiently described.

- How were missing data managed

- Include a discussion of multicollinearity testing.

- Quality ratings categorized as Poor/Fair, Good, and Excellent. Was this a self-reported single-item measurement? Was the scale validated? Was any psychometric testing performed?

DISCUSSION

Clarify differences between satisfaction, perceived quality, and actual quality.

Avoid overstating links between perception and mortality reduction unless directly supported by their data.

DATA AVAILABILITY

- This manuscript states that “all relevant data are within the manuscript and its Supporting Information files”. I will like to confirm “Is the full LARCHIS dataset publicly accessible?”.

- If it is available, a repository link to the data set or an access protocol number should be provided

Reviewer #3: The manuascipt in review titled “Quality of Maternal Health Services in Lagos, Nigeria: Women’s Perspectives Across Healthcare Districts” (Manuscript No. PONE-D-25-59599) addresses an important gap in maternal health services research by focusing on women’s perceptions of care quality across multiple healthcare districts in Lagos, Nigeria. The dataset is large, the analysis is largely sound, and the findings are relevant.

In terms of ccientific and public health value, the study uses a large, population based dataset (LARCHIS 2022; n = 7,324), which enhances statistical power and representativeness. There is a focus on patient reported quality of care, aligning with WHO’s emphasis on respectful, person centered maternal care, the manuscript has clear differentiation between structural factors (24 hour services, staffing, availability) and interpersonal factors (courtesy, responsiveness) and also analsysis district level comparisons which add important sub state granularity, which is valuable for policy and planning.

From a methods and analsysis perspective, the paper appropriately used of multinomial logistic regression for categorical outcomes (poor/fair, good, excellent) and includes mixed effects models to account for clustering by healthcare district is methodologically sound.

However, there are minor changes that should be addressed before publication as indicated below:

1. Ethics Statement – Clarification Required.

Issue: The manuscript submission metadata states “Ethics Statement: N/A”, while the Methods section later indicates that the study was approved by the Lagos State University Teaching Hospital Ethics Committee (LREC/06/10/1789) and involved written informed consent.

Recommendations: Correct the Ethics Statement in the submission system and manuscript to explicitly state the approving ethics committee, include the approval number, clarify consent procedures and anonymization, and remove “N/A” wherever it appears.

2. Data Availability Statement – Needs Precision

Issue: The manuscript states: “All relevant data are within the manuscript and its Supporting Information files.”

However, the analysis relies on LARCHIS 2022, which is a structured survey likely governed by data access rules.

Recomendatiions: Clarify whether LARCHIS data are publicly available. If so, provide repository name, URL, or access process. If not, explain third party ownership and access conditions and align Data Availability Statement with PLOS data policy language.

3. Abstract - Consider adding one quantitative result (e.g., strongest adjusted odds ratio) to strengthen impact.

4. Clarify Causal Language

Issue: The manuscript occasionally implies causality (e.g., “predictors,” “determine,” “lead to”), despite the cross sectional design.

Recommendation: Review language and tone down by consistently replacing causal phrasing with associational language: such as “associated with,” “correlated with,” “linked to”. In additiona, add one explicit sentence in the Discussion acknowledging that causality cannot be inferred.

Additioal suggested enhancement for discussions and interpretation: Expand briefly on why missionary/private facilities may outperform public ones beyond staffing (e.g., governance, accountability) and provide additional practical implications for Lagos State health system reform.

Reviewer #4: 1. The conclusions are made based on the findings and is scientifically sound.

2. The statistical analysis was done in a scientific manner. Both the bivariate and multivariate analysis were done to check the association of the different variables.

3. I said no because the dataset of the current manuscript is not findable to my search. however, if the PLOS editors are aware of this i will be convinced to change my answer to "YES"

4. The manuscript is written in English word.

Additional comments and feedbacks

1. Of the 44 participants excluded from the analysis of 10,000, one exclusion criterion was reporting a live birth that subsequently resulted in infant death. Nevertheless, the questionnaire included a reproductive health variable assessing infant loss, measured by the question: “Have you ever given birth to a child who was born alive but later died?” Based on this variable, 590 women reported experiencing infant loss. If the exclusion was actually due to missing/inconsistent responses that should be clarified, because otherwise it looks contradictory.

2. In the data management section what has been mentioned is how the primary data was managed. The dataset was screened for completeness and consistency, and the predefined inclusion and exclusion criteria were applied. However, additional data management steps undertaken for the current study shall be described.

**********

what does this mean?). If published, this will include your full peer review and any attached files.

If you choose “no”, your identity will remain anonymous but your review may still be made public.

**Do you want your identity to be public for this peer review?**  For information about this choice, including consent withdrawal, please see our Privacy Policy

Reviewer #1: **Yes:**  Friday Okonofua

Reviewer #2: No

Reviewer #3: **Yes:**  Oluwaseun Abel Aladesanmi

Reviewer #4: **Yes:**  tadele tesfean abraha

**********

[NOTE: If reviewer comments were submitted as an attachment file, they will be attached to this email and accessible via the submission site. Please log into your account, locate the manuscript record, and check for the action link "View Attachments". If this link does not appear, there are no attachment files.]

To ensure your figures meet our technical requirements, please review our figure guidelines: https://journals.plos.org/plosone/s/figures

You may also use PLOS’s free figure tool, NAAS, to help you prepare publication quality figures: https://journals.plos.org/plosone/s/figures#loc-tools-for-figure-preparation.

NAAS will assess whether your figures meet our technical requirements by comparing each figure against our figure specifications.

Attachment Submitted filename: PLOS ONE review December 2025(2) (1).docx

Attachment Submitted filename: Reviewer comment.pdf

# Author comment: Author response to Decision Letter 1

**DOI:** 10.1371/journal.pone.0359548.r002

1 Jul 2026

MANUSCRIPT #: PLOS ONE [PONE-D-25-59599]

Title: Quality of Maternal Health Services in Lagos, Nigeria: Women's Perspectives Across Healthcare Districts

Response to the Editor and Reviewers

We sincerely thank the Editor and Reviewers for their careful, thoughtful, and constructive evaluation of our manuscript, “Quality of Maternal Health Services in Lagos, Nigeria: Women’s Perspectives Across Healthcare Districts.” We are grateful for the opportunity to revise the manuscript and have carefully considered all comments. The feedback has substantially strengthened the rigor, clarity, and interpretability of our work. We have revised the manuscript to align closely with PLOS ONE editorial standards. Below, we provide detailed responses to each reviewer's comments.

Summarization of Edits from Reviews and Author Responses.

Reviewer #1

Comment #1: The quantitative design limits deeper insights into specific components of care quality. The responses simply categorized quality as poor, good, or excellent without identifying

components that were “good” or “excellent” to enable follow-up remediating actions to be

undertaken. I believe an add-on qualitative design or a more specific design, asking questions

on specific components of maternal care would have enabled the women to identify factors

that could be acted upon by policymakers to improve the quality of care in Lagos State.

However, I believe this point can be addressed as a limitation of the study in the discussion

section of the paper.

Response: We have expanded the Limitation section to explicitly acknowledge this limitation. We now emphasize that the LARCHIS dataset was not designed to capture granular dimensions of care quality, such as technical competence or interpersonal experience, and we highlight the need for future studies to use multi-item scale constructs to capture granular dimensions of maternal health care, mixed-methods studies, including qualitative approaches, to better identify actionable drivers of quality improvement from women’s perspectives. (Page 28).

Comment #2: The study date being 2022, the timing of previous maternity visits is unclear, making recall bias a real threat to the internal validity of the study.

Response: We have clarified in the Methods section that the LARCHIS survey was conducted in 2022 and collected retrospective data on recent maternal health experiences. We have also expanded the Discussion to explicitly acknowledge recall bias as a limitation, noting that while subjective perceptions may be affected, childbirth experiences are typically salient and therefore may be less prone to significant recall decay (page 28).

Comment #3: Novelty – There have been several publications on quality of maternal care in many parts of Nigeria, as well as other parts of Africa. The paper would do better if these past studies are summarized in a way to enable the identification of the specific novelty and research gaps being addressed by this study.

Response: We have substantially strengthened the Introduction by synthesizing the existing literature on maternal healthcare quality in Nigeria and sub-Saharan Africa. We now more clearly identify gaps in prior studies, including their reliance on facility-based or tertiary hospital data, limited use of population-based datasets, and lack of sub-state analyses. We also explicitly articulate the novelty of our study, particularly its use of a large, population-based dataset and its focus on district-level variation in women’s perceived quality of care. (page 7).

Comment # 4: Internal validity – The study uses an existing dataset from the 2022 – the Lagos Reproductive and Child Health Indicator Survey. More details about this dataset, including the data collection process and the quality, should be provided to enable readers see how the study

guide/protocol/questionnaire align with the specific objectives of this study.

Response: We have expanded the Methods section to provide a clearer description of the LARCHIS survey, including its sampling design, data collection procedures, and relevance to our research objectives. While we continue to cite the primary LARCHIS publication, we have ensured that sufficient detail is now included within the manuscript to allow readers to assess data quality and alignment with study aims. (page 8).

Comment #5: External validity – The limitations of the study and the implications of the results for policy and practice should be better discussed in the discussion section

Response: We have strengthened the Discussion to better address issues of external validity and policy relevance. We now discuss more clearly the generalizability of the findings to similar urban contexts and provide more detailed implications for policy and practice, particularly for improving person-centered care and addressing district-level disparities. We have also carefully reviewed the manuscript for typographical and grammatical errors and corrected these throughout.

Reviewer #2

We thank Reviewer #2 for their detailed and constructive feedback.

Comment #1: - The specific deficiencies in existing literature should be identified by this study. The significance of the problem to the targeted respondent needs to be better developed. The research gap is not clearly explained. More explanation on the gap that this study is willing to fill will be appreciated, and kindly highlight the primary and secondary research questions to be addressed

Response: We have substantially reorganized sections of the Introduction to improve clarity and logical flow. The revised Introduction now more clearly outlines deficiencies in the existing literature, strengthens the articulation of the public health significance of maternal healthcare quality in Lagos, and explicitly defines the research gap this study addresses. We have also added clear statements of the primary and secondary research objectives to guide the reader. (page 6).

Comment #2: According to the topic, you are looking at “women's perceptions of the quality of maternal health services” which they have received, not how barriers to access and perception of quality shape maternal health-seeking behavior. Kindly clarify this statement made in lines 118-120

Response: We have revised the relevant section to ensure that the manuscript consistently reflects that the study focuses on women’s perceptions of the quality of care received, rather than determinants of care-seeking behavior. Any references to health-seeking decisions are now framed as contextual background rather than study outcomes. (pages 6-7)

Comment #3: Explicit differentiation from previous analyses based on the Nigerian Demographic and Health Survey (NDHS) will be appreciated.

Response: We have added text explicitly noting in the Method section that our study is a secondary analysis of the LACHIS and NDHS datasets, which do not capture state-level granularity or specific factors such as newly created health districts in Lagos, as offered by LARCHIS. This distinction highlights the added value of our analysis in understanding district-level variation within Lagos. (page 8).

Comment #4: What is being referred to as formal health care system, as stated in line 114? As stated in line 112 to 114, does LARCHIS capture the reality of women who delivered outside hospital system? “Moreover, most existing research in Nigeria is derived from hospital-based studies, often at tertiary centers, which may not fully capture the realities of women who deliver outside formal healthcare systems”.

Response: We have clarified that “formal healthcare system” refers to recognized public and private health facilities staffed by trained providers, including primary, secondary, and tertiary institutions. We have also addressed the question of whether LARCHIS captures women delivering outside hospital settings by clarifying that while the dataset includes a range of facility types, it may underrepresent women who deliver entirely outside the formal healthcare system, which is now acknowledged as a limitation. (page 6)

Comment #5: - Dear author, the sentence in lines 120 to 122 looks vague and difficult to interpret; consider rephrasing the sentence so as to understand the context. “By situating maternal mortality within the paradox of Lagos, an urban megacity where abundant infrastructure coexists with poor maternal outcomes, this study seeks to identify structural and perceptual barriers driving healthcare decisions.”

Response: The previously unclear sentence regarding the “paradox of Lagos” has been rewritten to improve clarity and readability.

Comment #6: Provide detailed information on the weighting strategy and sampling methods. Inclusion criteria for the participants should be included. Line 137: Was it the time that the LARCHIS project was conducted that is being referred to here, or the date when your research was carried out? Kindly state explicitly when your research was done and when the LARCHIS data was collected as well.

Response: We have expanded the Methods section to provide detailed information on sampling procedures, weighting strategies, and inclusion criteria. We have clarified the data collection timeline (April-August 2022). (pages 8 and 9).

Comment #7: - Line 148, kindly add to the statement that no “new” participants were enrolled.

Response: We have stated in the manuscript that no new participants were enrolled in this secondary analysis. (page 9)

Comment #8: - STROBES guideline should be added. Analytic sample flow charts not visible in Figure 1

Response: We have added a statement confirming adherence to STROBE guidelines and included the checklist as supplementary material. The analytic sample flow chart (Figure 1) has been corrected to ensure visibility. (Page 10)

Comment #9: Under the “Ethics statement,” NA was written: this should be corrected, even though no new participants were used but it still involves human participants. And the “NA” that was filled contradicts your statement in lines 209 to 212.

Response: We appreciate the Reviewer’s attention to the inconsistency in the ethics statement. This has been fully corrected, with removal of “N/A” and inclusion of the ethics approval body, approval number, consent procedures, and data anonymization details.

Comment #10: - Model specification is insufficiently described.

Response: The Statistical Analysis section has been expanded to provide a more detailed description of model specification, including the use of multinomial regression and logistic mixed-effects modeling with the healthcare district specified as a random effect. (page 12)

Comment #11: - How were missing data managed

Response: We have added detailed information on how we handled missing data and how we obtained a final complete-case sample of 7,324 records, with at least 1,070 records per health district, which is large for the study. (Page 9 and Figure 1 on page 10)

Comment #12: - Include a discussion of multicollinearity testing.

Response: Below is the test for multicollinearity; no concerns were identified, as each GVI^(1/(2*Df)) is less than 2.

Comment #13: - Quality ratings categorized as Poor/Fair, Good, and Excellent. Was this a self-reported single-item measurement? Was the scale validated? Was any psychometric testing performed?

Response: The rating scale is a standard, widely validated single-item measure commonly used in population health studies, particularly in patient experience surveys to capture subjective evaluations of care. Usually, the scale is as follows: 1-Poor, 2-Fair, 3-Good, 4-Very Good, 5-Excellent. More details on the scale's validation can be found in the following studies: "Validating Patient Perspectives: A Study on the Reliability of Satisfaction Survey Tools" (https://pmc.ncbi.nlm.nih.gov/articles/PMC11881768/) and "Routine measurement of patient experience" (https://pmc.ncbi.nlm.nih.gov/articles/PMC9884896/). It does not require a validated psychometric scale because it is not a multi-item scale. However, we include it in the limitations and suggest that future studies use a multi-item scale to assess the granularity of maternal health care visit experiences.

Comment #14: Clarify differences between satisfaction, perceived quality, and actual quality.

Avoid overstating links between perception and mortality reduction unless directly supported by their data.

Response: We have clarified that the outcome variable was a self-reported single-item measure of perceived quality, a standard and commonly used public health survey item. In addition, we have revised the Discussion to clearly distinguish between perceived quality, patient satisfaction, and objective measures of care quality, and we have avoided overstating any links to maternal health outcomes.

Comment # 15: Is the full LARCHIS dataset publicly accessible? If it is available, a repository link to the data set or an access protocol number should be provided

Response: We have revised the Data Availability Statement to clarify that LARCHIS data are not fully publicly accessible and have provided a clear description of the data access process in accordance with PLOS ONE policy.

Reviewer #3

We thank Reviewer #3 for recognizing the scientific and public health value of this work and for the constructive suggestions provided. We also provide detailed responses to each comment below.

Comment #1: Ethics Statement – Clarification Required.

Response: We have corrected all inconsistencies by clearly stating the approving ethics committee, providing the approval number, and describing consent and anonymization procedures. All instances of “N/A” have been removed.

Comment #2: Data Availability Statement – Needs Precision

Response: We have also revised the Data Availability Statement to clearly specify data ownership and access procedures, ensuring alignment with PLOS ONE requirements. To strengthen the Abstract, we have included a key quantitative finding to enhance the impact and clarity of the study’s results.

Comment #3: Abstract - Consider adding one quantitative result (e.g., strongest adjusted odds ratio) to strengthen impact.

Response: We have revised the abstract by adding some quantitative results. (Page 3)

Comment #4: Clarify Causal Language

Response: We have carefully reviewed the manuscript and replaced causal terminology with appropriate language describing observational associations throughout. We have also added an explicit statement in the Discussion acknowledging that causal inferences cannot be drawn because of the cross-sectional nature of the study.

Comment #5: Additional suggested enhancement for discussions and interpretation: Expand briefly on why missionary/private facilities may outperform public ones beyond staffing (e.g., governance, accountability) and provide additional practical implications for Lagos State health system reform.

Response: We expanded the Discussion to elaborate on potential reasons why private and missionary facilities may outperform public facilities, including differences in governance, accountability, and patient-centered care practices. We have also strengthened the policy implications for health system reform in Lagos State. (Page 26)

Reviewer #4

We thank Reviewer #4 for their positive assessment of the study’s scientific rigor and relevance. We also provide detailed responses to each comment below

Comment #1: I said no because the dataset of the current manuscript is not findable to my search. however, if the PLOS editors are aware of this i will be convinced to change my answer to "YES"

Response: We have clarified the dataset's status and revised the Data Availability Statement to align with PLOS ONE policies.

Comment #2: Of the 44 participants excluded from the analysis of 10,000, one exclusion criterion was reporting a live birth that subsequently resulted in infant death. Nevertheless, the questionnaire included a reproductive health variable assessing infant loss, measured by the question: “Have you ever given birth to a child who was born alive but later died?” Based on this variable, 590 women reported experiencing infant loss. If the exclusion was actually due to missing/inconsistent responses, that should be clarified, because otherwise it looks contradictory.

Response: We have clarified that exclusions were due to missing or inconsistent responses rather than infant death per se, and we have revised the text to eliminate any ambiguity (see Figure 1).

Comment #3: In the data management section, what has been mentioned is how the primary data was managed. The dataset was screened for completeness and consistency, and the predefined inclusion and exclusion criteria were applied. However, additional data management steps undertaken for the current study shall be described.

Response: We have expanded the Data Management section to describe additional steps undertaken in this analysis, including data cleaning, variable recoding, consistency checks, and handling of missing data. This provides greater transparency regarding how the dataset was prepared for analysis. (Page 10)

Closing Statement

We again thank the Editor and Reviewers for their valuable feedback. The revisions have significantly improved the clarity, methodological rigor, and overall quality of the manuscript. We believe the manuscript is now substantially strengthened and meets the standards required for publication in PLOS ONE. We appreciate your consideration and look forward to your response.

Attachment Submitted filename: Response to Reviewers (2).docx

# Editor report: Decision Letter 1

**DOI:** 10.1371/journal.pone.0359548.r003

**Authors:** Ayodeji Oginni

23 Jul 2026

Dear Dr. Momodu,

Thank you for submitting your manuscript to PLOS ONE. After careful consideration, we feel that it has merit but does not fully meet PLOS ONE’s publication criteria as it currently stands. Therefore, we invite you to submit a revised version of the manuscript that addresses the points raised during the review process.

Please submit your revised manuscript by Sep 06 2026 11:59PM. If you will need more time than this to complete your revisions, please reply to this message or contact the journal office at plosone@plos.org. When you're ready to submit your revision, log on to https://www.editorialmanager.com/pone/ and select the 'Submissions Needing Revision' folder to locate your manuscript file.

- A letter that responds to each point raised by the academic editor and reviewer(s). You should upload this letter as a separate file labeled 'Response to Reviewers'.
- A marked-up copy of your manuscript that highlights changes made to the original version. You should upload this as a separate file labeled 'Revised Manuscript with Track Changes'.
- An unmarked version of your revised paper without tracked changes. You should upload this as a separate file labeled 'Manuscript'.

If you would like to make changes to your financial disclosure, please include your updated statement in your cover letter. Guidelines for resubmitting your figure files are available below the reviewer comments at the end of this letter.

If applicable, we recommend that you deposit your laboratory protocols in protocols.io to enhance the reproducibility of your results. Protocols.io assigns your protocol its own identifier (DOI) so that it can be cited independently in the future. For instructions see: https://journals.plos.org/plosone/s/submission-guidelines#loc-laboratory-protocols. Additionally, PLOS ONE offers an option for publishing peer-reviewed Lab Protocol articles, which describe protocols hosted on protocols.io. Read more information on sharing protocols at https://plos.org/protocols?utm_medium=editorial-email&utm_source=authorletters&utm_campaign=protocols.

As the corresponding author, your ORCID iD is verified in the submission system and will appear in the published article. PLOS supports the use of ORCID, and we encourage all coauthors to register for an ORCID iD and use it as well. Please encourage your coauthors to verify their ORCID iD within the submission system before final acceptance, as unverified ORCID iDs will not appear in the published article. *Only*  the individual author can complete the verification step; PLOS staff *cannot*  verify ORCID iDs on behalf of authors.

We look forward to receiving your revised manuscript.

Kind regards,

Ayodeji Babatunde Oginni

Academic Editor

PLOS One

Journal Requirements:

If the reviewer comments include a recommendation to cite specific previously published works, please review and evaluate these publications to determine whether they are relevant and should be cited. There is no requirement to cite these works unless the editor has indicated otherwise.

Please review your reference list to ensure that it is complete and correct. If you have cited papers that have been retracted, please include the rationale for doing so in the manuscript text, or remove these references and replace them with relevant current references. Any changes to the reference list should be mentioned in the rebuttal letter that accompanies your revised manuscript. If you need to cite a retracted article, indicate the article’s retracted status in the References list and also include a citation and full reference for the retraction notice.

[Note: HTML markup is below. Please do not edit.]

[NOTE: If reviewer comments were submitted as an attachment file, they will be attached to this email and accessible via the submission site. Please log into your account, locate the manuscript record, and check for the action link "View Attachments". If this link does not appear, there are no attachment files.]

To ensure your figures meet our technical requirements, please review our figure guidelines: https://journals.plos.org/plosone/s/figures

You may also use PLOS’s free figure tool, NAAS, to help you prepare publication quality figures: https://journals.plos.org/plosone/s/figures#loc-tools-for-figure-preparation.

NAAS will assess whether your figures meet our technical requirements by comparing each figure against our figure specifications.

# Author comment: Author response to Decision Letter 2

**DOI:** 10.1371/journal.pone.0359548.r004

2 Sep 2026

MANUSCRIPT #: PLOS ONE [PONE-D-25-59599]

Title: Quality of Maternal Health Services in Lagos, Nigeria: Women's Perspectives Across Healthcare Districts

Response to the Editor and Reviewers

We sincerely thank the Editor and Reviewers for their careful, thoughtful, and constructive evaluation of our manuscript, “Quality of Maternal Health Services in Lagos, Nigeria: Women’s Perspectives Across Healthcare Districts.” We have carefully considered the comments and have addressed the requested revisions. Below, we provide our response to each comment and indicate the corresponding changes made to the manuscript.

Summary of Authors’ Responses.

Academic Editor

Comment #1: Kindly revise the Abstract section to ensure that the word counts do not exceed 300 words.

Response: We have revised the Abstract to comply with the PLOS ONE requirement of no more than 300 words. The total word count of the abstract is now 269 words.

Comment #2: Please ensure that you refer to Table 2 in your text, as, if accepted, production will need this reference to link the reader to the Table.

Response: Table 2 is now referenced in the manuscript. (page 13).

Attachment Submitted filename: Response to Reviewers .docx

# Editor report: Decision Letter 2

**DOI:** 10.1371/journal.pone.0359548.r005

**Authors:** Ayodeji Oginni

15 Sep 2026

Quality of Maternal Health Services in Lagos, Nigeria: Women’s Perspectives Across Healthcare Districts

PONE-D-25-59599R2

Dear Dr. Momodu,

We’re pleased to inform you that your manuscript has been judged scientifically suitable for publication and will be formally accepted for publication once it meets all outstanding technical requirements.

Within one week, you’ll receive an e-mail detailing the required amendments. When these have been addressed, you’ll receive a formal acceptance letter and your manuscript will be scheduled for publication.

An invoice will be generated when your article is formally accepted. Please note, if your institution has a publishing partnership with PLOS and your article meets the relevant criteria, all or part of your publication costs will be covered. Please make sure your user information is up-to-date by logging into Editorial Manager at Editorial Manager® and clicking the ‘Update My Information' link at the top of the page. For questions related to billing, please contact billing support.

If your institution or institutions have a press office, please notify them about your upcoming paper to help maximize its impact. If they’ll be preparing press materials, please inform our press team as soon as possible -- no later than 48 hours after receiving the formal acceptance. Your manuscript will remain under strict press embargo until 2 pm Eastern Time on the date of publication. For more information, please contact onepress@plos.org.

Kind regards,

Ayodeji Babatunde Oginni

Academic Editor

PLOS One

Additional Editor Comments (optional):

Reviewers' comments:

# Editor report: Acceptance letter

**DOI:** 10.1371/journal.pone.0359548.r006

**Authors:** Ayodeji Oginni

PONE-D-25-59599R2

PLOS One

Dear Dr. Momodu,

I'm pleased to inform you that your manuscript has been deemed suitable for publication in PLOS One. Congratulations! Your manuscript is now being handed over to our production team.

At this stage, our production department will prepare your paper for publication. This includes ensuring the following:

* All references, tables, and figures are properly cited

* All relevant supporting information is included in the manuscript submission,

* There are no issues that prevent the paper from being properly typeset

You will receive further instructions from the production team, including instructions on how to review your proof when it is ready. Please keep in mind that we are working through a large volume of accepted articles, so please give us a few days to review your paper and let you know the next and final steps.

Lastly, if your institution or institutions have a press office, please let them know about your upcoming paper now to help maximize its impact. If they'll be preparing press materials, please inform our press team within the next 48 hours. Your manuscript will remain under strict press embargo until 2 pm Eastern Time on the date of publication. For more information, please contact onepress@plos.org.

You will receive an invoice from PLOS for your publication fee after your manuscript has reached the completed accept phase. If you receive an email requesting payment before acceptance or for any other service, this may be a phishing scheme. Learn how to identify phishing emails and protect your accounts at https://explore.plos.org/phishing.

If we can help with anything else, please email us at customercare@plos.org.

Thank you for submitting your work to PLOS One and supporting open access.

Kind regards,

PLOS One Editorial Office Staff

on behalf of

Ayodeji Babatunde Oginni

Academic Editor

PLOS One
